# Association of osteoporosis treatment with risk of fracture, cardiovascular disease, and all-cause mortality in patients on maintenance dialysis: a retrospective database study using real-world data in Japan

**DOI:** 10.1007/s10157-026-02858-1

**Published:** 2026-04-25

**Authors:** Yasuo Imanishi, Kanae Takahashi, Hisako Yoshida, Ryota Kawai, Yuki Eguchi, Kengo Saito, Yu Sadachi, Ayumi Shintani

**Affiliations:** 1 Department of Vascular Medicine, Vascular Science Center for Translational Research, Osaka Metropolitan University Graduate School of Medicine, 1-4-3 Asahimachi, Abeno-ku, Osaka, Japan; 2 Department of Medical Statistics, Osaka Metropolitan University Graduate School of Medicine, 1-4-3 Asahimachi, Abeno-ku, Osaka, Japan; 3 Primary Medical Science Department, Medical Affairs Division, Daiichi Sankyo Co., Ltd., 3-5-1 Nihonbashi Honcho, Chuo-ku, Tokyo, Japan; 4 Data Intelligence Department, Global DX, Daiichi Sankyo Co., Ltd., 1-2-58 Hiromachi, Shinagawa-ku, Tokyo, Japan

**Keywords:** Cardiovascular disease, Dialysis, Fracture, Mortality, Osteoporosis, Real-world evidence

## Abstract

**Background:**

Patients with chronic kidney disease who have progressed to dialysis treatment have increased fracture risk. However, the impact of osteoporosis treatment on fracture risk has not been evaluated in a large-scale study using Japanese real-world data.

**Methods:**

In this retrospective observational study (UMIN000054749), DeSC-IQVIA Integrated Claims Data were used to investigate the impact of osteoporosis treatment on fracture risk and other events in patients receiving maintenance hemo- or peritoneal dialysis. Data from April 2014 to August 2022 were extracted, and patients were divided into treated/untreated groups based on prescription records for osteoporosis medications during a 1-year exposure assessment period. The primary endpoint was the incidence of total and hip fractures from the index date (1 year post-exposure assessment period) until the end of follow-up.

**Results:**

Of 156,557 patients receiving maintenance dialysis for ≥ 1 year, 38,246 were included: 1093 and 37,153 in the treated and untreated groups, respectively. Although there was a numerically higher fracture incidence in the treated group, no significant difference was observed between the groups overall. As aged, the difference in fracture risk between the groups decreased. Multivariable regression analysis revealed that age, sex, fracture history (primary risk factor), diabetes, and sleep disorder were statistically significant effect modifiers of fracture risk.

**Conclusion:**

The numerically higher incidence of fractures in the treated group may have been due to patient background differences. Fracture risk management is essential in dialysis patients, and osteoporosis treatment should be considered at an earlier age, taking into account the patient’s background.

**Supplementary Information:**

The online version contains supplementary material available at 10.1007/s10157-026-02858-1.

## Introduction

Estimates suggest that approximately 13% of the adult Japanese population has chronic kidney disease (CKD) [1]. Dialysis treatment has been steadily increasing in Japan, from approximately 162/100,000 individuals in 2000 to 278/100,000 in 2022 [2].

Patients with CKD undergoing dialysis have an increased risk of musculoskeletal complications. Reduced kidney function elicits abnormalities in mineral metabolism and endocrine function, leading to aberrations in bone remodeling [3, 4]. Several studies have demonstrated that having CKD necessitating dialysis is associated with increased risks of osteoporosis and fragility fractures [5–8]. Bone abnormalities are therefore extremely common among patients with CKD, with nearly all stage G5 patients experiencing bone-related complications [3].

There are serious clinical issues of bone-related complications in CKD. Fragility fractures have a considerable impact on morbidity, mortality, and quality of life [9–11]. Preventive measures are essential for avoiding fragility fractures in patients undergoing dialysis. The recent European consensus statement on the therapeutic intervention and monitoring for osteoporosis and fragility fractures in patients with CKD stages G4–G5D provides guidance on risk assessment and pharmacological/non-pharmacological interventions [12]. However, in Japan, consensus has not been reached on appropriate management. Moreover, the implementation of bone mineral density (BMD) measurements and osteoporosis medication prescription status typical of actual clinical practice in Japan for CKD patients on dialysis have not been investigated in detail.

In addition to bone-related complications, patients with CKD on dialysis also have an elevated risk of cardiovascular disease as a result of vascular calcification, which manifests as CKD–mineral and bone disorder [4, 13, 14]. Although prevention of cardiovascular events and bone-related complications is important in reducing morbidity and mortality in patients with CKD [13, 15], there are few data regarding the impact of osteoporosis treatment on morbidity and mortality outcomes in patients receiving dialysis in Japan.

We conducted the present study to investigate the impact of osteoporosis treatment interventions on fracture risk, cardiovascular disease, all-cause mortality, and the use of bone densitometry in patients undergoing maintenance hemo- or peritoneal dialysis in Japan.

## Materials and methods

### Study design

This was a retrospective cohort study using health insurance claims data (DeSC-IQVIA Integrated Claims Data) licensed by IQVIA Solutions Japan G.K.; Tokyo, Japan [16]. To minimize bias, we selected this database because of its large size, comprising patients across a wide age range, including the advanced elderly. This database aggregates data from the three major independent payer systems in Japan: Social Insurance (used by full-time permanent company employees), National Health Insurance (used by non-full time/non-permanent employees or unemployed individuals), and the Medical Care System for the Advanced Elderly (which includes individuals aged ≥ 75 years or ≥ 65 years with a specific disability), making it one of the largest claims databases in Japan. The database includes anonymized data of both health insurance claims and diagnostic procedure combinations based on the International Classification of Diseases 10th Revision (ICD-10 codes), linking routinely collected information for each patient such as demographics, medical procedures, prescription records, and survival status.

**Fig. 1:**
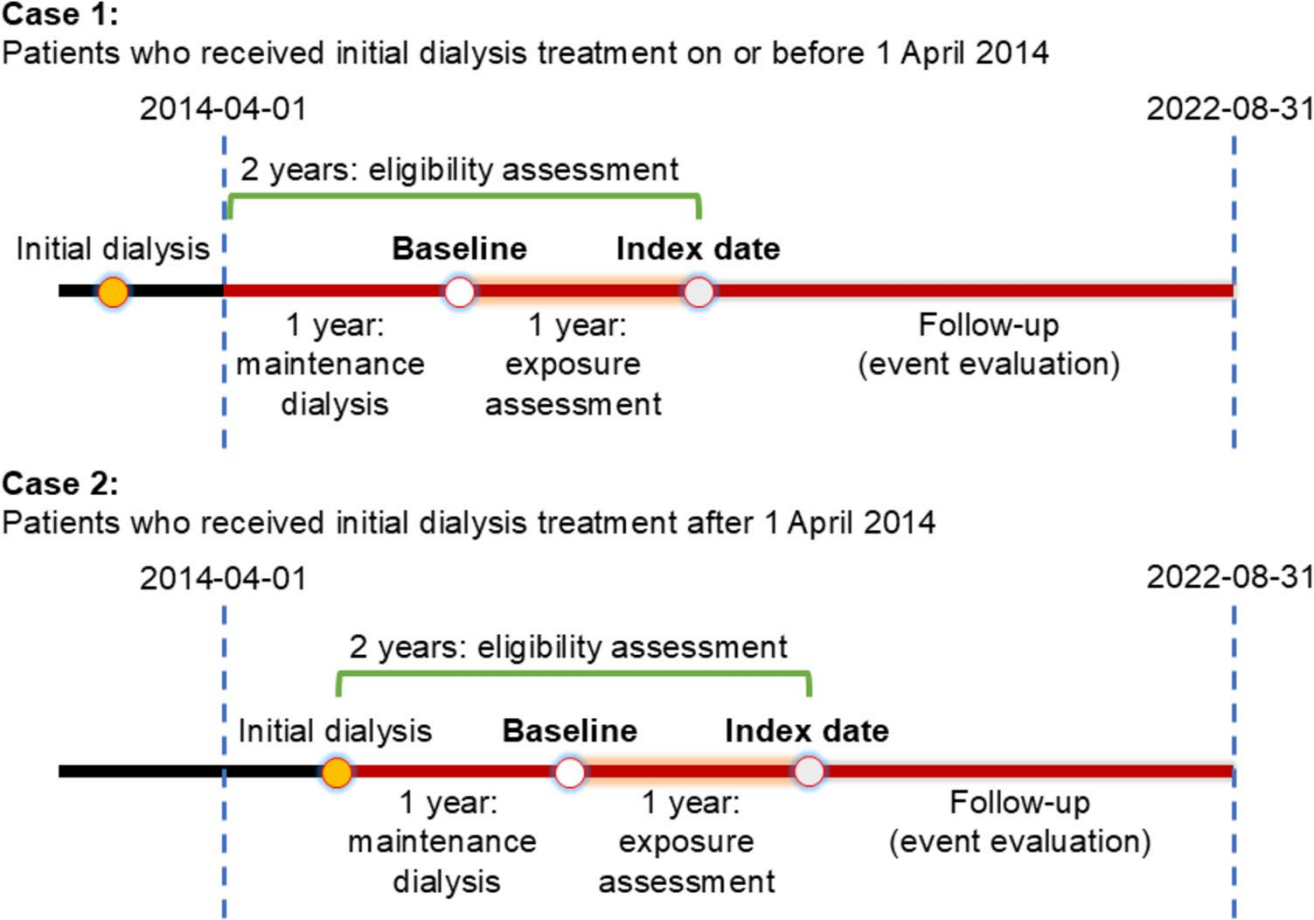
Study design. Patients were divided into “treated” and “untreated” groups. “Treated” refers to patients who had a prescription record for an osteoporosis medication during the exposure assessment period (between baseline and the index date). “Untreated” refers to patients with no prescription record for an osteoporosis medication during the exposure assessment period

The study design is illustrated in Fig. 1. Data from 1 April 2014 to 31 August 2022 were collected. Baseline was defined as the dialysis treatment date closest to 1 year after the initiation of dialysis, and the index date was defined as 1 year after baseline. The follow-up period began from the index date and continued until the earliest of the following occurred: last record of data in the database, death, or 31 August 2022 (study end).

Patients were classified into a treated group and an untreated group, based on whether they had a prescription record for an osteoporosis medication within 1 year prior to the index date (exposure assessment period). Study outcomes were evaluated during the follow-up period. Patients who received osteoporosis treatment were classified as the “treated” group, including patients with any prescription records for any of the following medications: denosumab, romosozumab, bisphosphonates, parathyroid hormone preparations, calcium preparations, or selective estrogen receptor modulators. A comprehensive list of the medications used to define this group is provided in Online Resource 1. Vitamin D preparations were not included among the osteoporosis medications used to define the treated group in this study, because treatment guidelines recommend monitoring vitamin D levels and supplementation for all patients with CKD undergoing dialysis [17].

### Ethics

This study was conducted per the principles of the Declaration of Helsinki (revised in 2013) and the Act on the Protection of Personal Information (Act No. 57 of 30 May 2003). The study was registered in the University Hospital Medical Information Network Clinical Trials Registry (identifier: UMIN000054749) on 1 July 2024. Because this was a retrospective analysis of claims data using fully anonymized data, the requirement for review by an ethics committee was waived, and informed consent was not required.

### Participants

The inclusion criteria were: undergoing maintenance hemodialysis or peritoneal dialysis procedures (identified by receipt codes for “artificial kidney” or “peritoneal perfusion”) for at least 2 consecutive years between 1 April 2014 and 31 August 2022, and age ≥ 20 years at baseline.

Patients were excluded from the study if any of the following applied: kidney transplantation after the initial dialysis treatment date but before the index date; presence of comorbidity associated with decreased BMD/prescription of osteoporosis medication prior to baseline; cancer diagnosis after the initial dialysis treatment date but before the index date; parathyroidectomy prior to the index date; or death prior to the index date. Comprehensive lists of ICD-10 codes that apply to the eligibility criteria are presented in Online Resources 2 and 3.

### Outcomes

The primary endpoint was the incidence of fractures (total fractures and hip fractures) after the index date. Total fractures was defined as the sum of all recorded fractures according to the ICD-10 codes listed in Online Resource 4.

The secondary endpoints included the rate of BMD measurements; osteoporosis and bone metabolism-related drug prescriptions; incidence of all-cause mortality; and incidence of cardiovascular disease (including atrial fibrillation, congestive heart failure, stroke, angina pectoris, and myocardial infarction) after the index date. ICD-10 codes for mortality and cardiovascular disease are also included in Online Resource 4. BMD measurements were identified by receipt codes for quantitative bone mineral analysis [lumbar spine by dual energy x-ray absorptiometry (DXA)] (160091310), bone mineral determination (microdensitometry, single energy x-ray absorptiometry, or other) (160147310), and quantitative bone mineral count (ultrasonography) (160170410).

### Statistical methods

Because this was an exploratory study, there was no prespecified sample size calculation. For baseline characteristics, frequencies and proportions were used to describe categorical variables. Patients were censored if they reached the last observed day after the index date or if the patients could not be followed due to changes in insurance coverage.

Between-group differences in fracture incidence were analyzed using the Fine–Gray method, with death as a competing risk [18]. The following covariates were used in the multivariable regression model to control for confounding: age at baseline, sex, comorbidities, incident dialysis initiation, calendar month, fracture history, and prescription drug history at the index date. Specific comorbidities and medications included as covariates are listed in Online Resource 5.

For assessing the effect of osteoporosis treatment over a wide age range, we conducted multivariable regression analyses, adjusting for the above-described covariates and a cross-product term between osteoporosis treatment and age at baseline. In the multivariable regression models, the restricted cubic spline function with five knots was applied to age at baseline to assess the non-linear effect.

For the outcome of all-cause death, the Cox proportional hazards model, adjusting for the above-described covariates, was used to calculate the hazard ratios (HRs) in the treated and untreated groups. The incidences of cardiovascular events in the treated and untreated groups were compared using the Fine–Gray method, where all-cause death was handled as a competing risk.

A two-sided 5% significance level was used for all statistical inferences, and no adjustments were made for multiple comparisons. All statistical analyses were conducted using R version 4.5.1 (The R Foundation for Statistical Computing, Vienna, Austria).

## Results

### Study population

The background characteristics of the patients are shown in Table 1. Data for a total of 156,557 patients receiving maintenance dialysis for ≥ 2 years were extracted from the database, of which 38,246 patients met the eligibility criteria and were included in the analysis. Of these, 1093 were classified in the treated group and 37,153 in the untreated group.

**Table 1:** Patient characteristics at baseline and up to the index date

	Treated^a^	Untreated^a^	Total
	*n* = 1093	*n* = 37,153	*N* = 38,246
Sex			
Male	425 (38.9)	25,142 (67.7)	25,567 (66.8)
Age (years)			
≥ 65	897 (82.1)	26,456 (71.2)	27,353 (71.5)
Age group (years)			
20 to < 25	0 (0.0)	16 (0.0)	16 (0.0)
25 to < 30	0 (0.0)	49 (0.1)	49 (0.1)
30 to < 35	2 (0.2)	161 (0.4)	163 (0.4)
35 to < 40	7 (0.6)	333 (0.9)	340 (0.9)
40 to < 45	9 (0.8)	829 (2.2)	838 (2.2)
45 to < 50	16 (1.5)	1422 (3.8)	1438 (3.8)
50 to < 55	41 (3.8)	2020 (5.4)	2061 (5.4)
55 to < 60	51 (4.7)	2670 (7.2)	2721 (7.1)
60 to < 65	70 (6.4)	3197 (8.6)	3267 (8.5)
65 to < 70	140 (12.8)	5604 (15.1)	5744 (15.0)
70 to < 75	180 (16.5)	5919 (15.9)	6099 (15.9)
75 to < 80	229 (21.0)	6480 (17.4)	6709 (17.5)
80 to < 85	181 (16.6)	4815 (13.0)	4996 (13.1)
85 to < 90	129 (11.8)	2711 (7.3)	2840 (7.4)
90 to < 95	34 (3.1)	821 (2.2)	855 (2.2)
95 to < 100	4 (0.4)	102 (0.3)	106 (0.3)
≥ 100	0 (0.0)	4 (0.0)	4 (0.0)
History of fracture			
Yes	512 (46.8)	5886 (15.8)	6398 (16.7)
Comorbidities			
Hypertension	1063 (97.3)	35,850 (96.5)	36,913 (96.5)
Dyslipidemia	632 (57.8)	20,077 (54.0)	20,709 (54.1)
Hyperuricemia	502 (45.9)	17,662 (47.5)	18,164 (47.5)
Diabetes	654 (59.8)	22,305 (60.0)	22,959 (60.0)
Alcoholism	3 (0.3)	48 (0.1)	51 (0.1)
Rheumatoid arthritis	64 (5.9)	1004 (2.7)	1068 (2.8)
Dementia	225 (20.6)	5978 (16.1)	6203 (16.2)
Sleep disorder	648 (59.3)	18,516 (49.8)	19,164 (50.1)
Chronic obstructive pulmonary disease	37 (3.4)	1345 (3.6)	1382 (3.6)
Concomitant medications			
Oral glucocorticoid	137 (12.5)	2562 (6.9)	2699 (7.1)
Proton pump inhibitor	844 (77.2)	25,045 (67.4)	25,889 (67.7)
Hormonal therapy	6 (0.5)	139 (0.4)	145 (0.4)
Thiazolidinedione	2 (0.2)	56 (0.2)	58 (0.2)
Oral beta-blocker	536 (49.0)	16,227 (43.7)	16,763 (43.8)
Oral loop diuretic	417 (38.2)	13,050 (35.1)	13,467 (35.2)
Warfarin	149 (13.6)	3968 (10.7)	4117 (10.8)
Anti-anxiety drug	645 (59.0)	17,772 (47.8)	18,417 (48.2)
Antiepileptic drug	97 (8.9)	2701 (7.3)	2798 (7.3)
Selective serotonin reuptake inhibitor	21 (1.9)	549 (1.5)	570 (1.5)
Secondary hyperparathyroidism treatment drug	1029 (94.1)	33,810 (91.0)	34,839 (91.1)
Calcimimetic	771 (70.5)	23,280 (62.7)	24,051 (62.9)
Phosphate binder	931 (85.2)	31,262 (84.1)	32,193 (84.2)

Data are* n* (%)

Sex and age data are as recorded at baseline. History of fractures, comorbidities, and concomitant medication information are those recorded during the time period from the date of initial dialysis treatment to the index date

^a^“Treated” refers to patients who had a prescription record for an osteoporosis medication during the exposure assessment period (between baseline and the index date). “Untreated” refers to patients with no prescription record for an osteoporosis medication during the exposure assessment period

Overall, 66.8% of patients were male, with a notable imbalance in sex between groups (38.9% and 67.7% of patients were male in the treated and untreated groups, respectively). Approximately 70% were ≥ 65 years old. A history of fractures was recorded in 46.8% of patients in the treated group and 15.8% in the untreated group; this was the characteristic with the greatest between-group difference.

The most common comorbidities were hypertension and diabetes, recorded in 97.3% and 59.8% of patients in the treated group, and 96.5% and 60.0% in the untreated group, respectively. Dyslipidemia (57.8% and 54.0%) and sleep disorder (59.3% and 49.8%) were also common. The most common concomitant medications included secondary hyperparathyroidism treatment drugs and proton pump inhibitors, recorded in 94.1% and 77.2% of patients in the treated group and 91.0% and 67.4% in the untreated group, respectively.

### Incidence of fracture

**Fig. 2:**
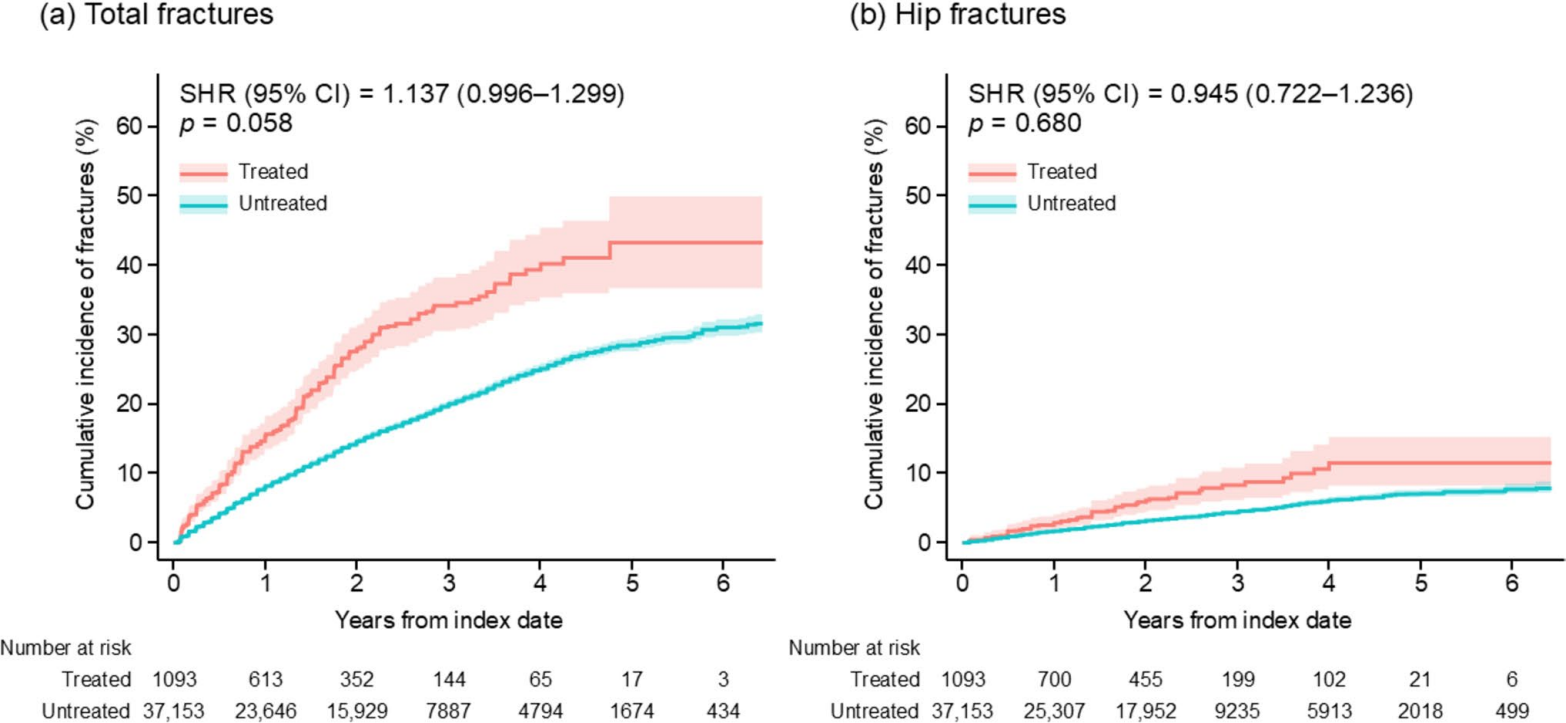
Cumulative fracture incidence for (**a**) total fractures, and (**b**) hip fractures in patients on maintenance dialysis with/without prescription records for osteoporosis medications. *CI* confidence interval, *SHR* sub-distribution hazard ratio

The cumulative incidences of total fractures in the treated and the untreated groups are shown in Fig. 2a. Although fracture rates were numerically higher in the treated group, the risk of fracture was not statistically significantly different between groups: sub-distribution HR (SHR) 1.137 (95% confidence interval [CI] 0.996–1.299), *p* = 0.058. Figure 2b shows the cumulative incidence of hip fractures in the treated and untreated groups. The risk of hip fracture was not significantly different: SHR 0.945 (95% CI 0.722–1.236), *p* = 0.680.

### Osteoporosis treatment vs non-treatment and fracture risk

#### Age and sex

**Fig. 3:**
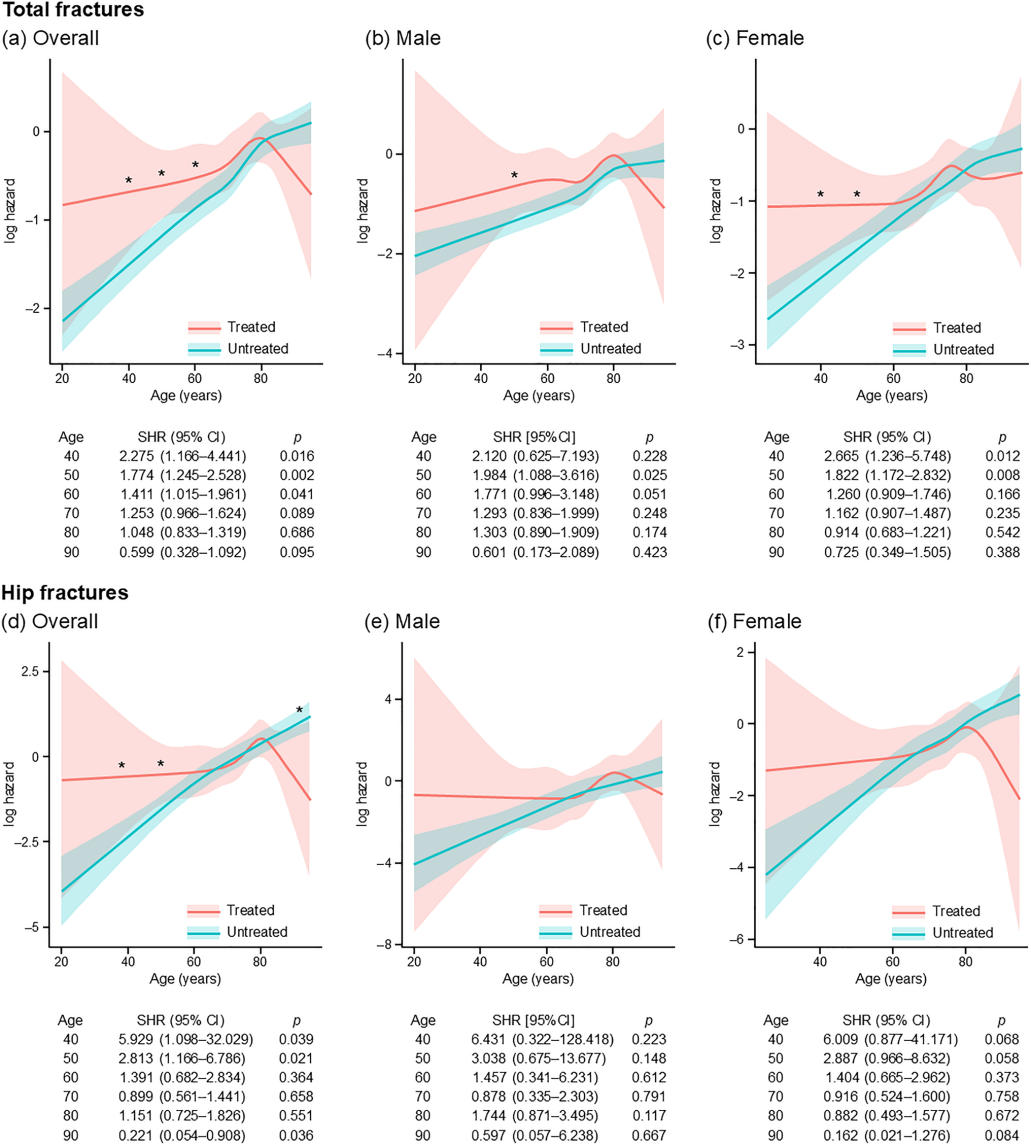
Risk of fracture by age group in patients on maintenance dialysis with/without prescription records for osteoporosis medications. (**a**) Total fracture risk in all patients; (**b**) total fracture risk in male patients; **c** total fracture risk in female patients; (**d**) hip fracture risk in all patients; (**e**) hip fracture risk in male patients; and (**f**) hip fracture risk in female patients. * indicates a significant difference between the treated and untreated groups. *CI* confidence interval, *SHR* sub-distribution hazard ratio

The multivariable regression analysis for total fracture risk at specific ages showed that the treated group had a higher risk of fracture vs the untreated group at ages 40, 50, and 60 years (SHR [95% CI] 2.275 [1.166–4.441], 1.774 [1.245–2.528], and 1.411 [1.015–1.961], respectively, all *p* < 0.05; Fig. 3a). There was no statistically significant difference in total fracture risk between treated and untreated patients aged ≥ 70 years.

Stratified by sex, statistically significant differences in total fracture risk were only identified in men aged 50 years and women aged 40 or 50 years, who were more likely to have fractures if they had received osteoporosis treatment (Fig. 3b and c). No significant differences in fracture risk according to osteoporosis treatment were shown for men or women aged ≥ 60 years.

The multivariable regression analysis for hip fracture risk showed that the treated group had a higher risk of hip fracture vs the untreated group at ages 40 and 50, with a SHR of 5.929 (95% CI 1.098–32.029; *p* = 0.039) and 2.813 (95% CI 1.166–6.786; *p* = 0.021; Fig. 3d). No statistically significant differences in hip fracture risk were identified by age between treated and untreated groups when stratified by sex (Fig. 3e and f).

#### Other background factors

Statistically significant patient background effect modifiers for both total fractures and hip fractures were age at baseline, sex, history of fracture, diabetes, and sleep disorder (*p* values all < 0.05; Table 2).

**Table 2:** Risk factors for total fractures and hip fractures

	Total fractures			Hip fractures		
	SHR	95% CI	*p* value	SHR	95% CI	*p* value
Osteoporosis treatment during the exposure assessment period	1.137	(0.996, 1.299)	0.058	0.945	(0.722, 1.236)	0.680
Age at baseline	1.033	(1.030, 1.036)	< 0.001	1.060	(1.054, 1.067)	< 0.001
Sex (male)	0.719	(0.679, 0.760)	< 0.001	0.626	(0.556, 0.705)	< 0.001
History of fracture	2.340	(2.202, 2.486)	< 0.001	2.627	(2.329, 2.964)	< 0.001
Hypertension	1.128	(0.964, 1.319)	0.130	0.852	(0.634, 1.144)	0.290
Dyslipidemia	0.985	(0.931, 1.041)	0.590	0.849	(0.755, 0.956)	0.007
Hyperuricemia	0.991	(0.937, 1.047)	0.750	0.963	(0.856, 1.084)	0.530
Diabetes	1.080	(1.020, 1.143)	0.008	1.346	(1.194, 1.519)	< 0.001
Alcoholism	1.856	(0.953, 3.611)	0.069	1.941	(0.478, 7.884)	0.350
Rheumatoid arthritis	1.079	(0.931, 1.251)	0.310	1.352	(1.015, 1.801)	0.039
Dementia	1.036	(0.963, 1.114)	0.350	1.092	(0.949, 1.257)	0.220
Sleep disorder	1.102	(1.019, 1.190)	0.014	1.294	(1.098, 1.525)	0.002
Chronic obstructive pulmonary disease	1.042	(0.902, 1.204)	0.580	1.325	(1.002, 1.752)	0.048
Oral glucocorticoid	1.149	(1.039, 1.271)	0.007	0.998	(0.798, 1.248)	0.990
Proton pump inhibitor	1.158	(1.090, 1.230)	< 0.001	1.093	(0.964, 1.240)	0.160
Hormonal therapy	1.423	(0.949, 2.133)	0.088	1.053	(0.390, 2.845)	0.920
Thiazolidinedione	0.759	(0.361, 1.597)	0.470	0.985	(0.247, 3.918)	0.980
Oral beta-blocker	1.061	(1.004, 1.121)	0.034	1.070	(0.954, 1.201)	0.250
Oral loop diuretic	1.073	(1.012, 1.138)	0.019	1.097	(0.969, 1.241)	0.140
Warfarin	0.984	(0.904, 1.070)	0.700	1.068	(0.902, 1.266)	0.440
Anti-anxiety drugs	1.061	(0.982, 1.147)	0.130	1.006	(0.855, 1.185)	0.940
Antiepileptic drugs	1.150	(1.041, 1.269)	0.006	1.107	(0.896, 1.368)	0.340
Selective serotonin reuptake inhibitor	0.776	(0.608, 0.989)	0.041	0.684	(0.399, 1.174)	0.170
Secondary hyperparathyroidism treatment drug	1.179	(1.020, 1.361)	0.025	1.065	(0.800, 1.417)	0.670
Calcimimetic	0.964	(0.907, 1.024)	0.230	0.916	(0.806, 1.042)	0.180
Phosphate binder	0.996	(0.897, 1.107)	0.940	1.000	(0.805, 1.242)	1.000

*CI* confidence interval, *SHR* sub-distribution hazard ratio

### Bone mineral density measurements and prescription osteoporosis medications

The proportions of BMD measurements after the index date were 60.0% and 39.5% in the treated and untreated groups, respectively (Table 3). Proportions according to other patient background factors are also shown in Table 3.

**Table 3:** Bone mineral density measurements after the index date by patient baseline factors

	Treated^a^		Untreated^a^		Total	
	*n*	Record of bone mineral density measurement^b^	*n*	Record of bone mineral density measurement^b^	*n*	Record of bone mineral density measurement^b^
Overall	1093	656 (60.0)	37,153	14,691 (39.5)	38,246	15,347 (40.1)
Sex						
Male	425	256 (60.2)	25,142	9821 (39.1)	25,567	10,077 (39.4)
Female	668	400 (59.9)	12,011	4870 (40.5)	12,679	5270 (41.6)
Age (years)						
20 to < 30	0	0 (0)	65	24 (36.9)	65	24 (36.9)
30 to < 40	9	7 (77.8)	494	234 (47.4)	503	241 (47.9)
40 to < 50	25	19 (76.0)	2251	1026 (45.6)	2276	1045 (45.9)
50 to < 60	92	60 (65.2)	4690	2215 (47.2)	4782	2275 (47.6)
60 to < 70	210	126 (60.0)	8801	3537 (40.2)	9011	3663 (40.7)
70 to < 80	409	242 (59.2)	12,399	4750 (38.3)	12,808	4992 (39.0)
80 to < 90	310	186 (60.0)	7526	2651 (35.2)	7836	2837 (36.2)
≥ 90	38	16 (42.1)	927	254 (27.4)	965	270 (28.0)
< 65	196	125 (63.8)	10,697	4770 (44.6)	10,893	4895 (44.9)
≥ 65	897	531 (59.2)	26,456	9921 (37.5)	27,353	10,452 (38.2)
History of fracture						
Yes	512	281 (54.9)	5886	2204 (37.4)	6398	2485 (38.8)
No	581	375 (64.5)	31,267	12,487 (39.9)	31,848	12,862 (40.4)

Data are *n* or *n* (%)

^a^“Treated” refers to patients who had a prescription record for an osteoporosis medication during the exposure assessment period (between baseline and the index date). “Untreated” refers to patients with no prescription record for an osteoporosis medication during the exposure assessment period

^b^Identified by receipt codes for quantitative bone mineral analysis (lumbar spine by dual energy x-ray absorptiometry), bone mineral determination (microdensitometry, single energy x-ray absorptiometry, or other), and quantitative bone mineral count (ultrasonography)

The proportions of osteoporosis medication prescriptions after the index date are shown in Table 4. In the treated group, 74.9% of patients were prescribed a medication for osteoporosis after the index date and 5.8% of patients in the untreated group were prescribed an osteoporosis medication after the index date. The most commonly prescribed drugs in the treated group during the follow-up period included bisphosphonates and the anti-receptor activator of nuclear factor-κB ligand (RANKL) drug denosumab (41.8% and 20.6%, respectively).

**Table 4:** Prescription osteoporosis medications after the index date

	Treated^a^ (*n* = 1093)	Untreated^a^ (*n* = 37,153)	Total (*N* = 38,246)
Osteoporosis and bone metabolism-related drugs	819 (74.9)	2152 (5.8)	2971 (7.8)
Selective estrogen receptor modulators	33 (3.0)	100 (0.3)	133 (0.3)
Bazedoxifene acetate	22 (2.0)	47 (0.1)	69 (0.2)
Raloxifene hydrochloride	14 (1.3)	56 (0.2)	70 (0.2)
Ipriflavone	1 (0.1)	4 (0.0)	5 (0.0)
Calcium drugs	21 (1.9)	96 (0.3)	117 (0.3)
Calcium hydrogen phosphate hydrate	0 (0.0)	2 (0.0)	2 (0.0)
Calcium l-aspartate hydrate	21 (1.9)	94 (0.3)	115 (0.3)
Calcitonin drugs	71 (6.5)	338 (0.9)	409 (1.1)
Elcatonin	71 (6.5)	338 (0.9)	409 (1.1)
Calcitonin (salmon)	0 (0.0)	1 (0.0)	1 (0.0)
Bisphosphonates	457 (41.8)	954 (2.6)	1411 (3.7)
Alendronate (PO)	109 (10.0)	282 (0.8)	391 (1.0)
Ibandronate (PO)	316 (28.9)	599 (1.6)	915 (2.4)
Minodronic acid (PO)	43 (3.9)	87 (0.2)	130 (0.3)
Risedronate (PO)	6 (0.5)	36 (0.1)	42 (0.1)
Zoledronate (IV)	1 (0.1)	0 (0.0)	1 (0.0)
Vitamin K_2_ drugs (menatetrenone)	46 (4.2)	127 (0.3)	173 (0.5)
Parathyroid hormone drugs	44 (4.0)	108 (0.3)	152 (0.4)
Teriparatide acetate	29 (2.7)	84 (0.2)	113 (0.3)
Teriparatide (genetic recombination)	15 (1.4)	26 (0.1)	41 (0.1)
Female hormone drugs	9 (0.8)	41 (0.1)	50 (0.1)
Estriol	7 (0.6)	25 (0.1)	32 (0.1)
Estradiol	1 (0.1)	9 (0.0)	10 (0.0)
Estradiol valerate	0 (0.0)	1 (0.0)	1 (0.0)
Conjugated estrogens	1 (0.1)	7 (0.0)	8 (0.0)
Anti-RANKL antibody (denosumab)	225 (20.6)	499 (1.3)	724 (1.9)
Anti-sclerostin antibody (romosozumab)	43 (3.9)	156 (0.4)	199 (0.5)

Data are *n* (%)

*IV* intravenous administration, *PO* oral administration, *RANKL* receptor activator of nuclear factor-κ B ligand

^a^“Treated” refers to patients who had a prescription record for an osteoporosis medication during the exposure assessment period (between baseline and the index date). “Untreated” refers to patients with no prescription record for an osteoporosis medication during the exposure assessment period

### Mortality and cardiovascular disease

**Fig. 4:**
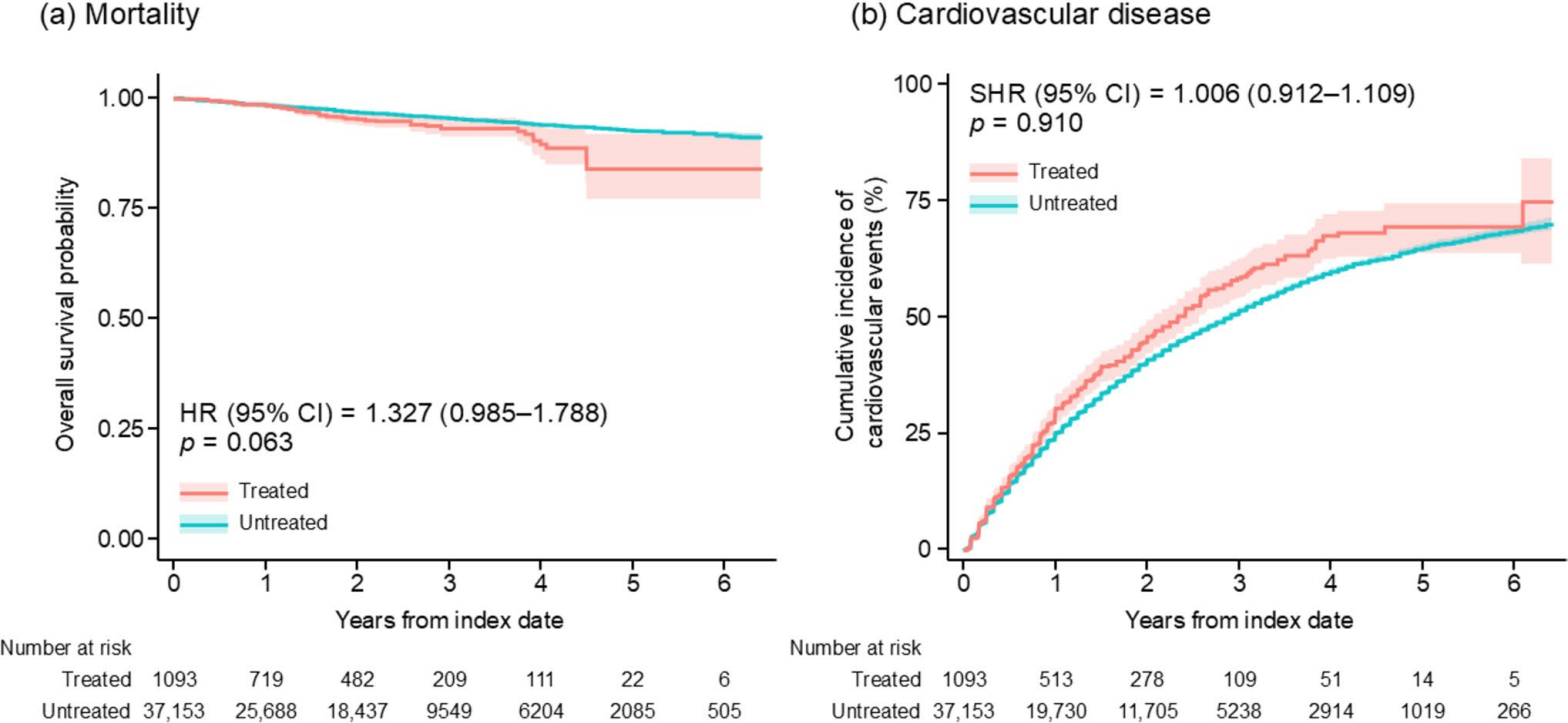
All-cause mortality (**a**) and cardiovascular disease (**b**) in patients on maintenance dialysis with/without prescription records for osteoporosis medications. *CI* confidence interval, *HR* hazard ratio, *SHR* sub-distribution HR

The incidence of all-cause mortality was numerically higher in the treated vs untreated group (4.3% and 3.2%, respectively), but the risk of death was not statistically significantly different between groups: HR 1.327 (95% CI 0.985–1.788, *p* = 0.063; Fig. 4a). The risks of cardiovascular events in the treated and untreated groups were similar (40.2% and 38.8%, respectively); SHR 1.006 (95% CI 0.912–1.109, *p* = 0.910; Fig. 4b).

Table 5 shows risk factors found to be associated with mortality or cardiovascular disease. Effect modifiers with the strongest association for mortality (*p* < 0.001) were age at baseline, sex, history of fracture, diabetes, dementia, and warfarin use. For cardiovascular disease, age at baseline, sex, history of fracture, dyslipidemia, hyperuricemia, diabetes, chronic obstructive pulmonary disease, proton pump inhibitors, beta-blockers, warfarin, and anti-anxiety drugs were most strongly associated (*p* < 0.001).

**Table 5:** Risk factors for mortality and cardiovascular disease

	Mortality			Cardiovascular disease		
	HR	95% CI	*p* value	SHR	95% CI	*p* value
Osteoporosis treatment during the exposure assessment period	1.327	(0.985, 1.788)	0.063	1.006	(0.912, 1.109)	0.910
Age at baseline	1.036	(1.031, 1.042)	< 0.001	1.020	(1.018, 1.021)	< 0.001
Sex (male)	1.628	(1.426, 1.859)	< 0.001	1.137	(1.097, 1.178)	< 0.001
History of fracture	1.354	(1.178, 1.557)	< 0.001	1.145	(1.096, 1.197)	< 0.001
Hypertension	0.930	(0.672, 1.288)	0.663	1.065	(0.968, 1.172)	0.200
Dyslipidemia	0.968	(0.860, 1.089)	0.588	1.131	(1.093, 1.171)	< 0.001
Hyperuricemia	0.976	(0.868, 1.097)	0.681	0.934	(0.902, 0.966)	< 0.001
Diabetes	1.688	(1.486, 1.917)	< 0.001	1.221	(1.178, 1.264)	< 0.001
Alcoholism	1.252	(0.311, 5.039)	0.751	1.142	(0.741, 1.759)	0.550
Rheumatoid arthritis	0.943	(0.667, 1.334)	0.741	1.162	(1.060, 1.274)	0.001
Dementia	1.309	(1.131, 1.515)	< 0.001	1.044	(0.996, 1.094)	0.071
Sleep disorder	1.095	(0.931, 1.288)	0.272	1.047	(0.999, 1.098)	0.057
Chronic obstructive pulmonary disease	1.324	(1.026, 1.708)	0.031	1.226	(1.126, 1.335)	< 0.001
Oral glucocorticoid	1.085	(0.874, 1.347)	0.458	1.103	(1.036, 1.175)	0.002
Proton pump inhibitor	1.246	(1.093, 1.421)	0.001	1.295	(1.248, 1.344)	< 0.001
Hormonal therapy	2.100	(1.046, 4.217)	0.037	0.954	(0.728, 1.250)	0.730
Thiazolidinedione	0.835	(0.208, 3.349)	0.799	1.733	(1.259, 2.386)	0.001
Oral beta-blocker	1.207	(1.075, 1.354)	0.001	1.336	(1.293, 1.382)	< 0.001
Oral loop diuretic	1.023	(0.904, 1.158)	0.721	1.051	(1.013, 1.089)	0.008
Warfarin	1.402	(1.201, 1.637)	< 0.001	1.335	(1.271, 1.402)	< 0.001
Anti-anxiety drugs	1.055	(0.897, 1.242)	0.517	1.106	(1.055, 1.160)	< 0.001
Antiepileptic drugs	1.032	(0.837, 1.273)	0.766	1.075	(1.010, 1.144)	0.023
Selective serotonin reuptake inhibitor	1.312	(0.865, 1.990)	0.201	1.019	(0.893, 1.163)	0.780
Secondary hyperparathyroidism treatment drug	0.992	(0.743, 1.325)	0.957	1.010	(0.921, 1.108)	0.830
Calcimimetic	1.073	(0.941, 1.224)	0.291	1.048	(1.010, 1.088)	0.014
Phosphate binder	0.854	(0.692, 1.055)	0.143	1.026	(0.959, 1.097)	0.460

*CI* confidence interval, *HR* hazard ratio, *SHR* sub-distribution HR

## Discussion

In this retrospective, observational database study, we investigated the association of osteoporosis treatment with fracture risk, cardiovascular disease, mortality, and use of BMD measurement in Japanese patients on maintenance dialysis for CKD. To our knowledge, ours is the first large-scale analysis to examine the relationship between osteoporosis treatment and fracture incidence in patients receiving maintenance dialysis in Japan. Notably, there was a higher incidence of fracture history in the treated vs untreated group (46.8% vs 15.8%); the treated group also had more women (61.1% vs 32.3%) and older age (≥ 65 years, 82.1% vs 71.2%).

We hypothesized that the untreated group would have a higher incidence of both total fractures and hip fractures than the treated group, and that the untreated group would also have a higher incidence of cardiovascular events and all-cause mortality. However, this was not the case. Although the treated group had a numerically higher incidence of fractures than the untreated group, the difference was not statistically significant, and a similar result was observed for cardiovascular events and all-cause mortality. It should be noted that the number of patients in the treated group was quite small, and the treated group had higher proportions of patients with history of fracture, those aged ≥ 65 years, and women. Although these factors were statistically adjusted for, we speculate that these factors nevertheless introduced bias that may have influenced the fracture rate in the treated group, thus obscuring the effect of treatment. Indeed, previous research has identified prior fracture [19], female sex [20], and older age [21] as being associated with increased fracture risk, consistent with the findings of our study.

We found that the incidence of total fractures in both men and women aged ≥ 60 years, and the incidence of hip fractures in both men and women aged ≥ 40 years, was not significantly different between treated and untreated groups. Based on these results, we anticipate that even among dialysis patients who are not prescribed osteoporosis treatment, the fracture risk would become indistinguishable from that of treated patients as they age. Therefore, regardless of age, it is important to consider continuous BMD measurements to assess bone status, as well as treatment with osteoporosis medications for fracture prevention.

The BMD measurement rate among all patients in our study was approximately 40%, which was higher than that reported previously for stage G3–5 CKD patients not receiving dialysis (5.3%) [22]. However, this likely reflects the fact that patients on dialysis are prone to osteoporosis [3, 4], and physicians are therefore more likely to recommend patients undergo BMD screening. Nevertheless, a screening rate of only 40% may be too low for patients receiving dialysis, and this could be a result of the relatively low prescription rates of osteoporosis medications in this study: 2.9% up to the index date and 7.8% during the follow-up period.

Measuring BMD to detect osteoporosis at an early stage is critical for improving patient prognosis and quality of life [23]. To gain a better understanding of the condition of individual patients, we expect that tests other than BMD measurement will be useful in guiding treatment decisions in clinical practice. Although not collected in this study, bone quality data are also important for assessing overall bone health [24], and assessments of bone turnover markers are essential in this regard. Moreover, blood sampling offers a simpler, more accessible route to monitoring bone health than DXA scanning, which is not available at all healthcare facilities.

Among physicians performing dialysis treatment, osteoporosis and fracture risk/prevention may not have received sufficient attention. Moreover, among patients who were prescribed osteoporosis medications, there were cases in which drugs contraindicated for dialysis patients were prescribed, such as calcium l-aspartate hydrate, risedronate, and zoledronate. Although treatment intervention for osteoporosis is important for improving patient prognosis, the selection of therapeutic drugs with consideration of patient safety is also extremely important. In the future, osteoporosis treatment for patients on dialysis is expected to become more common; however, further evidence, including data on fracture risk reduction, is needed to optimize therapeutic strategies.

Finally, because of the high mortality rate and risk of cardiovascular disease in dialysis patients with a history of fractures, comprehensive care for fracture prevention, focusing on overall mortality and cardiovascular disease risk, is expected to be a future challenge. For dialysis patients, managing risk factors such as dyslipidemia, hyperuricemia, diabetes, rheumatoid arthritis, and chronic obstructive pulmonary disease are critically important.

The limitations of this study included the following. Owing to the large discrepancy in patient numbers between the treated and untreated groups, it was not possible to apply propensity score matching techniques to reduce bias between the groups. Statistical testing for between-group differences in patient background factors was not prespecified in the study protocol; therefore, potential bias with respect to background factors in the groups could not be confirmed. Approximately 6% of individuals in the untreated group received prescriptions for osteoporosis medication after the index date, and approximately 25% of individuals in the treated group did not receive prescriptions for osteoporosis medications after the index date, which likely introduced confounding. A small proportion of the ICD-10 codes do not specify the fracture site, which may have introduced error in the number of site-specific (hip) fractures. Finally, because data for cardiovascular disease as a pre-existing condition or comorbidity at baseline were not collected, the relationship between the baseline presence of these factors and subsequent occurrence of cardiovascular disease is unclear.

In conclusion, the osteoporosis-treated group had a higher incidence of fractures than the untreated group. Even among dialysis patients in the untreated group, as they aged, there was no longer a clear difference in fracture risk compared with dialysis patients in the treated group. Even in patients considered to have a low fracture risk, it is necessary to consider appropriate fracture risk management and the introduction of osteoporosis treatment according to the level of risk, regardless of patient age. Given that a history of fracture is a risk factor for death and cardiovascular disease, it is important to implement more rigorous osteoporosis management for dialysis patients with a history of fractures, as well as fracture prevention measures for those without a history of fractures.

## Supplementary Information

Below is the link to the electronic supplementary material.

Supplementary file1 (DOCX 101 KB)

## Data Availability

The aggregated participant data underlying the results presented in this manuscript may be made available to researchers upon submission of a reasonable request to the corresponding author. The decision to disclose the data will be made by the corresponding author and the funder, Daiichi Sankyo Co., Ltd. Researchers who make the request should include a methodologically sound proposal on how the data will be used; the proposal may be reviewed by the responsible personnel at Daiichi Sankyo Co., Ltd., and the data requestors will need to sign a data access agreement. Data disclosure can be requested for 36 months from article publication. Please directly contact the corresponding author to request access to data.
