# Supplementary material for: Association of osteoporosis treatment with risk of fracture, cardiovascular disease, and all-cause mortality in patients on maintenance dialysis: a retrospective database study using real-world data in Japan
Source: Clin Exp Nephrol. 2026 Apr 25;30(7):1125–37. doi: 10.1007/s10157-026-02858-1 (PMC13291016; doi:10.1007/s10157-026-02858-1)
Supplement: Supplementary file 1 — Supplementary file1 (DOCX 101 KB) [file 10157_2026_2858_MOESM1_ESM.docx]

**Online Resources**

**Association of osteoporosis treatment with risk of fracture, cardiovascular disease, and all-cause mortality in patients on maintenance dialysis: a retrospective database study using real-world data in Japan**

*Clinical and Experimental Nephrology*

Yasuo Imanishi,***** Kanae Takahashi, Hisako Yoshida, Ryota Kawai, Yuki Eguchi, Kengo Saito, Yu Sadachi, Ayumi Shintani

***Corresponding author**

Department of Vascular Medicine, Vascular Science Center for Translational Research, Osaka Metropolitan University Graduate School of Medicine, 1-4-3 Asahimachi, Abeno-ku, Osaka, Japan

E-mail: imanishig@gmail.com

**Online Resource 1.** ATC codes used to identify osteoporosis treatment prescriptions and define the “treated” group in the present study

| **ATC** | **Generic name** | **Receipt name** |
| --- | --- | --- |
| A12A0 | Dibasic Calcium Phosphate Hydrate | Calcium hydrogenphosphate YI |
| A12A0 | Dibasic Calcium Phosphate Hydrate | Calcium Hydrogen Phosphate Ebis |
| A12A0 | Dibasic Calcium Phosphate Hydrate | Dibasic Calcium Phosphate Hydrate |
| A12A0 | Dibasic Calcium Phosphate Hydrate | Calcium hydrogen phosphate hydrate Yoshida |
| A12A0 | Dibasic Calcium Phosphate Hydrate | Yamazen's secondary phosphorus ash |
| A12A0 | Calcium L-aspartate hydrate | Aspara-CA Tablets 200 |
| A12A0 | Calcium L-aspartate hydrate | Ca L-Aspartate Tablets 200 mg "Sawai" |
| A12A0 | Calcium L-aspartate hydrate | ELSPRY CA Tablets 200 mg |
| A12A0 | Calcium L-aspartate hydrate | Calcium L-Aspartate Tablets 200 mg "Towa" |
| G03C0 | Estradiol | Dibigel 1 mg |
| G03C0 | Estradiol | Estradiol Adhesive 0.72 mg "F" 9 cm^2^ |
| G03C0 | Estradiol | Estrana tape 0.72 mg 9 cm^2^ |
| G03C0 | Estradiol | Julina Tablets 0.5 mg |
| G03C0 | Estradiol | Le Estrogel 0.06% |
| G03C0 | Estradiol | Estrana tape 0.09 mg 1.125 cm^2^ |
| G03C0 | Estradiol | Estrana tape 0.18 mg 2.25 cm^2^ |
| G03C0 | Estradiol | Estrana tape 0.36 mg 4.5 cm^2^ |
| G03C0 | Estradiol | Estrana 0.72 mg 9 cm^2^ |
| G03C0 | Estradiol propionate | Ovahormone Depot IM Injection 5 mg |
| G03C0 | Estradiol propionate | Ovahormone Depot 5mg |
| G03C0 | Estradiol valerate | Pelanin Depot 5 mg IM |
| G03C0 | Estradiol valerate | Pelanin Depot 10 mg IM |
| G03C0 | Estradiol valerate | Proginon Depot 10 mg IM |
| G03C0 | Estriol | Merrilac Tablets 1 mg |
| G03C0 | Estriol | Estriol Tablets 1mg "Department drugs" |
| G03C0 | Estriol | Estrile Tablets 100γ 0.1 mg |
| G03C0 | Estriol | Estrile Tablets 1mg (Mochida) |
| G03C0 | Estriol | Hollin Tablets 1 mg |
| G03C0 | Estriol | Estrile Tablets 0.5 mg |
| G03C0 | Estriol | Estriol Tablets 0.1 mg |
| G03C0 | Estriol | Estriol Tablets 0.5 mg |
| G03C0 | Estriol | Estriol Tablets 1 mg |
| G03C0 | Estriol | Estriol Tablets 1mg "F" |
| G03C0 | Estriol | Estriol Aqueous Suspension 10 mg 1 mL |
| G03C0 | Estriol propionate | Estrile Depot Injection 10 mg 1 mL |
| G03C0 | Fosfestrol | Honban Tablets 100 mg |
| G03C0 | Conjugated estrogens | Premarin Tablets 0.625 mg |
| G03J0 | Bazedoxifene Acetate | Viviant Tablets 20 mg |
| G03J0 | Bazedoxifene Acetate | Bazedoxifen Tablets 20 mg "Sawai" |
| G03J0 | Raloxifene hydrochloride | Evista Tablets 60 mg |
| G03J0 | Raloxifene hydrochloride | Raloxifene Hydrochloride Tablets 60 mg "Sawai" |
| G03J0 | Raloxifene hydrochloride | Raloxifene Hydrochloride Tablets 60 mg "Teva" |
| G03J0 | Raloxifene hydrochloride | Raloxifene Hydrochloride Tablets 60 mg "KN" |
| G03J0 | Raloxifene hydrochloride | Raloxifene Hydrochloride Tablets 60 mg "EE" |
| G03J0 | Raloxifene hydrochloride | Raloxifene Hydrochloride Tablets 60 mg "Towa" |
| G03J0 | Raloxifene hydrochloride | Raloxifene Hydrochloride Tablets 60 mg "SN" |
| G03J0 | Raloxifene hydrochloride | Raloxifene Hydrochloride Tablets 60 mg "Ayumi" |
| G03J0 | Raloxifene hydrochloride | Raloxifene Hydrochloride Tablets 60 mg "DK" |
| G03J0 | Raloxifene hydrochloride | Raloxifene Hydrochloride Tablets 60 mg "Nissin" |
| G03J0 | Raloxifene hydrochloride | Raloxifene Hydrochloride Tablets 60 mg "NICHIKO" |
| H04A0 | Elcatonin | Ercitonin Injection 20S Disposable 20 Elcatonin Units 1mL |
| H04A0 | Elcatonin | Eskatonyl for IM Injection 20 units 20 elcatonin units 1mL |
| H04A0 | Elcatonin | Erindacin Injection 20 units Elcatonin 20 units 1mL |
| H04A0 | Elcatonin | Osteonin Injection 20 Elcatonin Units 1mL |
| H04A0 | Elcatonin | Posevin Injection 20S 20 Elcatonin Units 1mL |
| H04A0 | Elcatonin | Laskarton Injection 20 Elcatonin Units 1mL |
| H04A0 | Elcatonin | Eridacin Injection 40 units Elcatonin 40 mL 1mL |
| H04A0 | Elcatonin | Elcatonin 20 Elcatonin Units 1mL injection |
| H04A0 | Elcatonin | Ercitonin Injection 40 units Elcatonin 40 mL 1mL |
| H04A0 | Elcatonin | Elka Intramuscular Injection 10 U 10 elcatonin 1 mL |
| H04A0 | Elcatonin | Elcatonin Injection 40 U "Takeda Teva" 40 Elcatonin U 1mL |
| H04A0 | Elcatonin | Elcatonin Injection 40 Units "TBP" 40 Elcatonin Units 1mL |
| H04A0 | Elcatonin | Elcatonin Injection 40 Units "Isai" 40 Elcatonin Units 1mL |
| H04A0 | Elcatonin | Elcatonin Injection 40 U "F" 40 Elcatonin U 1mL |
| H04A0 | Elcatonin | Elcatonin Injection 40 Units "NP" 40 Elcatonin Units 1mL |
| H04A0 | Elcatonin | Elcatonin Muscle Injection 10 U "Takeda Teva" 10 Elcatonin U 1mL |
| H04A0 | Elcatonin | Elcatonin Injection 10 U "TBP" 10 Elcatonin U 1mL |
| H04A0 | Elcatonin | Elcatonin Injection 10 U "Sawai" 10 Elcatonin U 1mL |
| H04A0 | Elcatonin | Elcatonin Intramuscular Injection 10 Units "Nichi-Iko" 10 Elcatonin Units 1mL |
| H04A0 | Elcatonin | Elcatonin IM 10 U "F" 10 Elcatonin U 1mL |
| H04A0 | Elcatonin | Elcatonin IM Injection 10 Units "NP" 10 Elcatonin Units 1mL |
| H04A0 | Elcatonin | Elcatonin Muscle Injection 20 U "Takeda Teva" 20 Elcatonin U 1mL |
| H04A0 | Elcatonin | Elcatonin Muscle Injection 20 Units "Nichi-Iko" 20 Elcatonin Units 1mL |
| H04A0 | Elcatonin | Elcatonin Muscle Injection 20 U "TOWA" 20 Elcatonin U 1mL |
| H04A0 | Elcatonin | Elcatonin 20 U IM "TBP" 20 Elcatonin U 1mL |
| H04A0 | Elcatonin | Elcatonin Myocardial Injection 20 U "F" 20 Elcatonin U 1mL |
| H04A0 | Elcatonin | Elcatonin Muscle Injection 20 U "Sawai" 20 Elcatonin U 1mL |
| H04A0 | Elcatonin | Elcatonin Muscle Injection 20 U "NP" 20 Elcatonin U 1mL |
| H04A0 | Elcatonin | Elcatonin 40 Elcatonin Units 1mL Injection |
| H04A0 | Elcatonin | Elcatonin 40 Elcatonin Units 1mL Injection |
| H04A0 | Elcatonin | Adeviroc Injection 40 Elcatonin 40 (1 mL) |
| H04A0 | Elcatonin | Osteonin Injection 10 Elcatonin Units 1mL |
| H04A0 | Elcatonin | Osteonin Injection 40 Elcatonin Units 1mL |
| H04A0 | Elcatonin | Echocartonin Injection 40 Elcatonin Units 1mL |
| H04A0 | Elcatonin | Laskarton 10 Disposable 10 Elcatonin Units 1mL |
| H04A0 | Elcatonin | Elcatonin 10 Elcatonin Units 1mL Injection |
| H04A0 | Elcatonin | Ercitonin Injection 10 units 10 elcatonin units 1mL |
| H04A0 | Elcatonin | Laskarton Injection 40 Elcatonin Units 1mL |
| H04A0 | Elcatonin | Laskarton Injection 10 Elcatonin Units 1mL |
| H04A0 | Elcatonin | Erindacin Injection 10 units Elcatonin 10 mL 1mL |
| H04A0 | Elcatonin | Posebin Injection 10 Elcatonin Units 1mL |
| H04A0 | Elcatonin | Ercitonin Injection 20S 20 Elcatonin Units 1mL |
| H04A0 | Calcitonin (salmon) | Thermotonin Intramuscular Injection 10 IU 0.5 mL |
| H04A0 | Calcitonin (salmon) | Calcitran Injection 10 1 mL in 10 IU |
| H04E0 | Abaloparatide acetate | Ostabaro Subcutaneous Injection Cartridge 1.5 mg 0.75 mL |
| H04E0 | Abaloparatide acetate | Ostabaro Subcutaneous Injection Cartridge 1.5mg 0.75mL once |
| H04E0 | Teriparatide Acetate | Teribone Inj. 56.5 μg |
| H04E0 | Teriparatide Acetate | Teribone for s.c. Injection 56.5 μg (with diluent) |
| H04E0 | Teriparatide Acetate | Teribone for s.c. injection 28.2 μg Autoinjector |
| H04E0 | Teriparatide Acetate | Teripalatide for s.c. Injection 56.5 μg "Sawai" (with diluent) |
| H04E0 | Teriparatide (Genetical Recombination) | Forteo s.c. injection kit 600 μg |
| H04E0 | Teriparatide (Genetical Recombination) | Forteo s.c. Injection Kit 600 μg 20 μg once |
| H04E0 | Teriparatide (genetical recombination) [teriparatide followed by 1] | Teripalatide BS Subcutaneous Injection Kit 600 μg "Motida" |
| H04E0 | Teriparatide (genetical recombination) [teriparatide followed by 1] | Teripalatide BS Subcutaneous Injection Kit 600 μg "Motida" 20 μg once |
| M05B3 | Alendronate sodium hydrate | Fosamac Tablets 55 mg |
| M05B3 | Alendronate sodium hydrate | Bonalon Tablets 5 mg |
| M05B3 | Alendronate sodium hydrate | Fosamac Tablets 35 mg |
| M05B3 | Alendronate sodium hydrate | Bonalon Tablets 35 mg |
| M05B3 | Alendronate sodium hydrate | Alendronate Tablets 5 mg "SN" |
| M05B3 | Alendronate sodium hydrate | Alendronate Tablets 5 mg "Taiyo" |
| M05B3 | Alendronate sodium hydrate | Alendronate Tablets 5 mg "DK" |
| M05B3 | Alendronate sodium hydrate | Alendronate Tablets 5 mg "Teva" |
| M05B3 | Alendronate sodium hydrate | Alendronate Tablets 5 mg "NIG" |
| M05B3 | Alendronate sodium hydrate | Alendronate Tablets 5 mg "Mylan" |
| M05B3 | Alendronate sodium hydrate | Alendronate Tablets 5 mg "Pfizer" |
| M05B3 | Alendronate sodium hydrate | Alendronate Tablets 5 mg "VTRS" |
| M05B3 | Alendronate sodium hydrate | Alendronate Tablets 5 mg "Amel" |
| M05B3 | Alendronate sodium hydrate | Alendronate Tablets 35 mg "Amel" |
| M05B3 | Alendronate sodium hydrate | Alendronate Tablets 35 mg "Taiyo" |
| M05B3 | Alendronate sodium hydrate | Alendronate Tablets 35 mg "Teva" |
| M05B3 | Alendronate sodium hydrate | Alendronate Tablets 35 mg "NIG" |
| M05B3 | Alendronate sodium hydrate | Alendronate Tablets 5 mg "Towa" |
| M05B3 | Alendronate sodium hydrate | Alendronate Tablets 35 mg "Towa" |
| M05B3 | Alendronate sodium hydrate | Alendronate Tablets 35 mg "Mylan" |
| M05B3 | Alendronate sodium hydrate | Alendronate Tablets 35 mg Pfizer |
| M05B3 | Alendronate sodium hydrate | Alendronate Tablets 35 mg "VTRS" |
| M05B3 | Alendronate sodium hydrate | Alendronate Tablets 35 mg "DK" |
| M05B3 | Alendronate sodium hydrate | Alendronate Tablets 5mg "F" |
| M05B3 | Alendronate sodium hydrate | Alendronate Tablets 35 mg "F" |
| M05B3 | Alendronate sodium hydrate | Alendronate Tablets 35 mg "SN" |
| M05B3 | Alendronate sodium hydrate | Alendronate Tablets 5 mg "Nichi-Iko" |
| M05B3 | Alendronate sodium hydrate | Alendronate Tablets 35 mg "Nichi-Iko" |
| M05B3 | Alendronate sodium hydrate | Bonalon Intravenous Drip Infusion Bag 900μg 100mL |
| M05B3 | Alendronate sodium hydrate | Alendronate Tablets 5 mg "YD" |
| M05B3 | Alendronate sodium hydrate | Alendronate Tablets 35 mg "YD" |
| M05B3 | Alendronate sodium hydrate | Alendronate Tablets 5 mg "TCK" |
| M05B3 | Alendronate sodium hydrate | Alendronate Tablets 35 mg "TCK" |
| M05B3 | Alendronate sodium hydrate | Bonalone Oral Jelly 35 mg |
| M05B3 | Alendronate sodium hydrate | Alendronate Tablets 5 mg "Sawai" |
| M05B3 | Alendronate sodium hydrate | Alendronate Tablets 35 mg Sawai |
| M05B3 | Alendronate sodium hydrate | Alendronate Tablets 5 mg "JG" |
| M05B3 | Alendronate sodium hydrate | Alendronate Tablets 35 mg "JG" |
| M05B3 | Alendronate sodium hydrate | Alendronate Tablets 5 mg "RTO" |
| M05B3 | Alendronate sodium hydrate | Alendronate Tablets 35 mg "RTO" |
| M05B3 | Alendronate sodium hydrate | Alendronate Intravenous Drip Infusion Bag 900 μg "HK" 100 mL |
| M05B3 | Alendronate sodium hydrate | Alendronate Intravenous Drip Infusion Bag 900 μg "DK" 100 mL |
| M05B3 | Alendronate sodium hydrate | Alendronate sodium 35 mg tablets |
| M05B3 | Alendronate sodium hydrate | Alendronate Sodium 5 mg tablets |
| M05B3 | Ibandronate sodium hydrate | Bombyva Intravenous Injection 1 mg Syringe 1 mL |
| M05B3 | Ibandronate sodium hydrate | Bombyva Tablets 100 mg |
| M05B3 | Ibandronate sodium hydrate | Ibandronate Intravenous Injection 1mg Syringe "HK" 1mL |
| M05B3 | Ibandronate sodium hydrate | Ibandronate Intravenous Injection 1mg Syringe "VTRS" 1mL |
| M05B3 | Ibandronate sodium hydrate | Ibandronate Intravenous Injection 1mg Syringe "Towa" 1mL |
| M05B3 | Ibandronate sodium hydrate | Ibandronate Intravenous Injection 1mg Syringe "Sawai" 1mL |
| M05B3 | Etidronate disodium | Dydronel Tablets 200 mg |
| M05B3 | Zoledronic acid | Reclast Intravenous Infusion 5 mg 100 mL |
| M05B3 | Zoledronic acid | Reclast Intravenous Infusion 5 mg 100 mL |
| M05B3 | Minodronic Acid Hydrate | Bonoteo Tablets 1mg |
| M05B3 | Minodronic Acid Hydrate | Ricarboxylic Acid Tablets 1 mg |
| M05B3 | Minodronic Acid Hydrate | Bonoteo Tablets 50 mg |
| M05B3 | Minodronic Acid Hydrate | RICARBON Tablets 50 mg |
| M05B3 | Minodronic Acid Hydrate | Minodronic acid Tablets 1mg "YD" |
| M05B3 | Minodronic Acid Hydrate | Minodronic acid Tablets 50 mg "YD" |
| M05B3 | Minodronic Acid Hydrate | Minodronic acid Tablets 1mg "Towa" |
| M05B3 | Minodronic Acid Hydrate | Minodronic Acid Tablets 50 mg "Towa" |
| M05B3 | Minodronic Acid Hydrate | Minodronic Acid Tablets 1mg "NICHIKO" |
| M05B3 | Minodronic Acid Hydrate | Minodronic Acid Tablets 50 mg "NICHIKO" |
| M05B3 | Minodronic Acid Hydrate | Minodronic Acid Tablets 1mg "JG" |
| M05B3 | Minodronic Acid Hydrate | Minodronic Acid Tablets 50 mg "JG" |
| M05B3 | Minodronic Acid Hydrate | Minodronic Acid Tablets 1mg Mikasa |
| M05B3 | Minodronic Acid Hydrate | Minodronic Acid Tablets 50 mg Mikasa |
| M05B3 | Minodronic Acid Hydrate | Minodronic Acid Tablets 50 mg "Ayumi" |
| M05B3 | Minodronic Acid Hydrate | Minodronic Acid Tablets 1mg "Sawai" |
| M05B3 | Minodronic Acid Hydrate | Minodronic Acid Tablets 50 mg "Sawai" |
| M05B3 | Minodronic Acid Hydrate | Minodronic Acid Tablets 1mg "Takeda Teva" |
| M05B3 | Minodronic Acid Hydrate | Minodronic acid Tablets 1mg "NIG" |
| M05B3 | Minodronic Acid Hydrate | Minodronic Acid Tablets 50 mg Takeda Teva |
| M05B3 | Minodronic Acid Hydrate | Minodronate Tablets 50 mg "NIG" |
| M05B3 | Minodronic Acid Hydrate | Minodronic acid Tablets 1mg "Nipro" |
| M05B3 | Minodronic Acid Hydrate | Minodronic Acid Tablets 50 mg "Nipro" |
| M05B3 | Minodronic Acid Hydrate | Minodronic acid 50 mg tablets |
| M05B3 | Minodronic Acid Hydrate | Minodronic acid 1 mg tablets |
| M05B3 | Sodium risedronate hydrate | Actonel Tablets 2.5 mg |
| M05B3 | Sodium risedronate hydrate | Benet Tablets 2.5 mg |
| M05B3 | Sodium risedronate hydrate | Actonel Tablets 17.5 mg |
| M05B3 | Sodium risedronate hydrate | Benet Tablets 17.5 mg |
| M05B3 | Sodium risedronate hydrate | Sodium risedronate tablets 2.5 mg "Meiji" |
| M05B3 | Sodium risedronate hydrate | Ricedronate Na Tablets 2.5 mg "Takata" |
| M05B3 | Sodium risedronate hydrate | Sodium Ricedronate Tablets 2.5 mg "Sawai" |
| M05B3 | Sodium risedronate hydrate | Sodium Ricedronate Tablets 2.5 mg "Tanabe" |
| M05B3 | Sodium risedronate hydrate | Sodium risedronate tablets 2.5 mg "ZE" |
| M05B3 | Sodium risedronate hydrate | Sodium risedronate tablets 2.5 mg "FFP" |
| M05B3 | Sodium risedronate hydrate | Sodium risedronate 2.5 mg "Nisshin" |
| M05B3 | Sodium risedronate hydrate | Sodium risedronate tablets 2.5 mg "Mylan" |
| M05B3 | Sodium risedronate hydrate | Sodium risedronate tablets 2.5 mg "Teva" |
| M05B3 | Sodium risedronate hydrate | Sodium Ricedronate Tablets 2.5 mg "Towa" |
| M05B3 | Sodium risedronate hydrate | Sodium risedronate tablets 2.5 mg "Chemiphar" |
| M05B3 | Sodium risedronate hydrate | Sodium risedronate tablets 2.5 mg "Utuc" |
| M05B3 | Sodium risedronate hydrate | Sodium risedronate tablets 2.5 mg "NP" |
| M05B3 | Sodium risedronate hydrate | Ricedronate Na Tablets 2.5 mg "Nichi-Iko" |
| M05B3 | Sodium risedronate hydrate | Sodium risedronate 2.5 mg "Pfizer" |
| M05B3 | Sodium risedronate hydrate | Sodium risedronate tablets 2.5 mg "F" |
| M05B3 | Sodium risedronate hydrate | Sodium risedronate 2.5 mg "JG" |
| M05B3 | Sodium risedronate hydrate | Sodium risedronate tablets 17.5 mg "Pfizer" |
| M05B3 | Sodium risedronate hydrate | Sodium risedronate tablets 17.5 mg "VTRS" |
| M05B3 | Sodium risedronate hydrate | Sodium risedronate tablets 17.5 mg "Sawai" |
| M05B3 | Sodium risedronate hydrate | Benet Tablets 75 mg |
| M05B3 | Sodium risedronate hydrate | Actonel Tablets 75 mg |
| M05B3 | Sodium risedronate hydrate | Sodium risedronate tablets 17.5 mg "Takata" |
| M05B3 | Sodium risedronate hydrate | Ricedronate Na Tablets 17.5 mg "Meiji" |
| M05B3 | Sodium risedronate hydrate | Sodium Ricedronate Tablets 17.5 mg "Nichi-Iko" |
| M05B3 | Sodium risedronate hydrate | Sodium risedronate tablets 17.5 mg "YD" |
| M05B3 | Sodium risedronate hydrate | Sodium risedronate tablets 2.5 mg "Kyorin" |
| M05B3 | Sodium risedronate hydrate | Sodium risedronate tablets 17.5 mg "Kyorin" |
| M05B3 | Sodium risedronate hydrate | Sodium risedronate tablets 17.5 mg "Towa" |
| M05B3 | Sodium risedronate hydrate | Sodium Ricedronate Tablets 17.5 mg "Amel" |
| M05B3 | Sodium risedronate hydrate | Sodium risedronate tablets 17.5 mg "ZE" |
| M05B3 | Sodium risedronate hydrate | Sodium risedronate tablets 17.5 mg "Teva" |
| M05B3 | Sodium risedronate hydrate | Sodium Ricedronate Tablets 17.5 mg "Tanabe" |
| M05B3 | Sodium risedronate hydrate | Sodium risedronate tablets 17.5 mg "Nisshin" |
| M05B3 | Sodium risedronate hydrate | Sodium risedronate tablets 2.5 mg "Sand" |
| M05B3 | Sodium risedronate hydrate | Sodium risedronate tablets 17.5 mg "Sand" |
| M05B3 | Sodium risedronate hydrate | Sodium risedronate tablets 17.5 mg "FFP" |
| M05B3 | Sodium risedronate hydrate | Sodium risedronate tablets 17.5 mg "F" |
| M05B3 | Sodium risedronate hydrate | Sodium risedronate tablets 17.5 mg "Chemiphar" |
| M05B3 | Sodium risedronate hydrate | Sodium risedronate tablets 17.5 mg "JG" |
| M05B3 | Sodium risedronate hydrate | Sodium risedronate tablets 17.5 mg "NP" |
| M05B3 | Sodium risedronate hydrate | Sodium risedronate tablets 17.5 mg "Utuc" |
| M05B3 | Sodium risedronate hydrate | Sodium risedronate tablets 17.5 mg "SN" |
| M05B3 | Sodium risedronate hydrate | Sodium risedronate 17.5 mg tablets |
| M05B3 | Sodium risedronate hydrate | Ricedronate Na Tablets 75 mg "Nichi-Iko" |
| M05B3 | Sodium risedronate hydrate | Sodium Ricedronate Tablets 75 mg "Towa" |
| M05B3 | Sodium risedronate hydrate | Sodium risedronate 75 mg tablets |
| M05B9 | Ipriflavone | Oricock Fine Granules 40% |
| M05B9 | Ipriflavone | Osten Tablets 200 mg |
| M05B9 | Ipriflavone | Ipriflavone 200 mg tablets |
| M05B9 | Ipriflavone | Cypoline Tablets 200 mg |
| M05B9 | Ipriflavone | Ipliflavone Tablets 200 mg "Taiyo" |
| M05B9 | Ipriflavone | Ipliflavone Fine Granules 40% "Taiyo" |
| M05B9 | Ipriflavone | Ipliflavone Tablets 200 mg "Cruhala" |
| M05B9 | Ipriflavone | Ipliflavone Tablets 200 mg "Sawai" |
| M05B9 | Ipriflavone | Ipliflavone Tablets 200 mg "Nichi-Iko" |
| M05B9 | Ipriflavone | Ipriflavone Tablets 200 mg "YD" |
| M05B9 | Ipriflavone | Ipliflavone Tablets 200 mg "Teva" |
| M05B9 | Ipriflavone | Ipriflavone 200 mg tablets |
| M05B9 | Denosumab (genetical recombination) | Pralia Subcutaneous Injection 60 mg Syringe 1 mL |
| M05B9 | Menatetrenone | Glakay Capsules 15 mg |
| M05B9 | Menatetrenone | Menatetrenone Capsules 15 mg "SN" |
| M05B9 | Menatetrenone | Menatetrenone Capsules 15 mg "F" |
| M05B9 | Menatetrenone | Menatetrenone Capsules 15 mg "Kaken" |
| M05B9 | Menatetrenone | Menatetrenone Capsules 15 mg "KTB" |
| M05B9 | Menatetrenone | Menatetrenone Capsules 15 mg "TCK" |
| M05B9 | Menatetrenone | Menatetrenone Capsules 15 mg "TYK" |
| M05B9 | Menatetrenone | Menatetrenone Capsules 15 mg "Organs of Japan" |
| M05B9 | Menatetrenone | Menatetrenone Capsules 15 mg "YD" |
| M05B9 | Menatetrenone | Menatetrenone Capsules 15 mg "Towa" |
| M05B9 | Menatetrenone | Menatetrenone Capsules 15 mg "Nichi-Iko" |
| M05B9 | Menatetrenone | Menatetrenone Capsules 15 mg "TC" |
| M05B9 | Menatetrenone | Menatetrenone Capsules 15 mg Tanabe |
| M05B9 | Menatetrenone | Menatetrenone Capsules 15 mg "CH" |
| M05B9 | Menatetrenone | Menatetrenone 15 mg Capsules |
| M05B9 | Lomosozumab (genetical recombination) | Ivenity Injection 105 mg Syringe 1.17 mL |

ATC, Anatomical Therapeutic Chemical classification

**Online Resource 2.** International Classification of Diseases 10^th^ Revision (ICD-10) codes relevant to the present study for inclusion criteria.

| **Dialysis diagnosis** | |  |
| --- | --- | --- |
| **ICD-10 code** | **ICD-10 name** | **Disease name** |
| N185 | Chronic kidney disease, stage 5 | Under chronic renal failure and maintenance dialysis |
| Z992 | Renal dialysis dependence | Chronic kidney disease stage G5D |
| Z992 | Renal dialysis dependence | Under chronic renal failure and maintenance dialysis |
| Z992 | Renal dialysis dependence | Renal dialysis status |
| **Dialysis treatment** | | |
| **ICD-10 Code** | **Receipt name** | |
| C102 | Home-based self-peritoneal drainage instruction and management fee | |
| C102-2 | Home hemodialysis guidance and management fee | |
| J0381 | Artificial kidneys (chronic maintenance dialysis) (<4 hours) | |
| J0381 | Artificial kidneys (chronic maintenance dialysis) (not less than 4 hours but less than 5 hours) | |
| J0381 | Artificial kidneys (chronic maintenance dialysis) (5 hours or longer) | |
| J0381 | Artificial kidney (chronic maintenance dialysis 1) (less than 4 hours) (specified patient) | |
| J0381 | Artificial kidney (chronic maintenance dialysis 1) (4 hours or more and less than 5 hours) (specified patient) | |
| J0381 | Artificial kidney (chronic maintenance dialysis 1) (5 hours or longer) (specified patient) | |
| J0381 | Artificial kidneys (chronic maintenance dialysis 1) (less than 4 hours) (interim measures) | |
| J0381 | Artificial kidneys (Chronic maintenance dialysis 1) (4 hours or more and less than 5 hours) (interim measures) | |
| J0381 | Artificial kidneys (Chronic maintenance dialysis 1) (5 hours or more) (interim measures) | |
| J0381 | Artificial kidneys (Chronic maintenance dialysis 1) (less than 4 hours) (excluding (a)) | |
| J0381 | Artificial kidneys (Chronic maintenance dialysis 1) (4 hours or more and less than 5 hours) (excluding b) | |
| J0381 | Artificial kidneys (chronic maintenance dialysis 1) (5 hours or longer) (excluding (c)) | |
| J03811 | Chronic maintenance dialysis filtration addition (artificial kidney) | |
| J0382 | Artificial kidneys (chronic maintenance dialysis filtration) (complex) | |
| J0382 | Artificial kidney (chronic maintenance dialysis 2) (less than 4 hours) (specified patient) | |
| J0382 | Artificial kidney (chronic maintenance dialysis 2) (4 hours or more and less than 5 hours) (specified patient) | |
| J0382 | Artificial kidney (chronic maintenance dialysis 2) (5 hours or longer) (specified patient) | |
| J0382 | Artificial kidneys (chronic maintenance dialysis 2) (less than 4 hours) (excluding (a)) | |
| J0382 | Artificial kidneys (chronic maintenance dialysis 2) (4 hours or more and less than 5 hours) (excluding b) | |
| J0382 | Artificial kidneys (chronic maintenance dialysis 2) (5 hours or longer) (excluding (c)) | |
| J0383 | Artificial kidney (chronic maintenance dialysis 3) (less than 4 hours) (specified patient) | |
| J0383 | Artificial kidney (chronic maintenance dialysis 3) (4 hours or more and less than 5 hours) (specified patient) | |
| J0383 | Artificial kidney (chronic maintenance dialysis 3) (5 hours or longer) (specified patient) | |
| J0383 | Artificial kidneys (chronic maintenance dialysis 3) (less than 4 hours) (excluding (a)) | |
| J0383 | Artificial kidneys (chronic maintenance dialysis 3) (4 hours or more and less than 5 hours) (excluding b) | |
| J0383 | Artificial kidneys (chronic maintenance dialysis 3) (5 hours or longer) (excluding (c)) | |
| J0384 | Artificial kidneys (others) | |
| J0384 | Addition of artificial kidney (induction period) | |
| J0386 | Additional amount in the run-in period 1 (artificial kidneys) | |
| J0386 | Additional amount in the run-in period 2 (artificial kidneys) | |
| J0421 | Continuous ambulatory peritoneal flow | |
| J0421 | Additional amount for the run-in period (peritoneal flow) | |
| J0422 | Abdominal flow of peritoneum (others) | |
| K610-3 | Internal shunt placement | |
| K610-3 | External shunt placement | |
| K6121 | Peripheral arteriovenous fistula (internal shunt construction) (simple) | |
| K6121 | Peripheral arteriovenous fistula (internal shunt), with venous dislocation | |
| K635-3 | Continuous ambulatory intraperitoneal catheterization | |

**Online Resource 3.** International Classification of Diseases 10^th^ Revision (ICD-10) codes relevant to the present study for exclusion criteria.

| **Kidney transplant** | |  |
| --- | --- | --- |
| **ICD-10 code** | **ICD-10 name** | **Disease name** |
| T856 | Other Specified Mechanical Complications of Internal Prostasis, Inserts and Grafts | CAPD catheter malposition |
| T856 | Other Specified Mechanical Complications of Internal Prostasis, Inserts and Grafts | CAPD catheter fracture |
| T857 | Other in vivo prosthesis, infections with inserts and grafts and inflammatory reactions | CAPD catheter tunnel infection |
| T857 | Other in vivo prosthesis, infections with inserts and grafts and inflammatory reactions | CAPD exit site infection |
| T857 | Other in vivo prosthesis, infections with inserts and grafts and inflammatory reactions | CAPD peritonitis |
| T861 | Renal transplant failure and rejection | Renal tubulointerstitial damage in transplant rejection |
| T861 | Renal transplant failure and rejection | Acute renal transplant rejection |
| T861 | Renal transplant failure and rejection | Kidney transplant rejection |
| T861 | Renal transplant failure and rejection | Kidney transplant failure |
| T861 | Renal transplant failure and rejection | Chronic renal transplant rejection |
| Z940 | Condition after kidney transplantation | Prolonged hyperparathyroidism |
| Z940 | Condition after kidney transplantation | After cadaveric kidney transplantation |
| Z940 | Condition after kidney transplantation | After kidney transplantation |
| Z940 | Condition after kidney transplantation | After living donor kidney transplantation |
| Z940 | Condition after kidney transplantation | After brain-dead kidney transplantation |
| **Kidney transplant (treatment)** | |  |
| **ICD-10 code** |  | **Receipt name** |
| K709-5 |  | Allogeneic cadaveric pancreas kidney transplantation |
| K780 |  | Allogeneic cadaveric kidney transplantation |
| K7801 |  | Additional transplant donation (allogeneic cadaveric kidney transplant) |
| K7802 |  | Additional fee for anti-HLA antibody test (allogeneic cadaveric kidney transplantation) |
| K780-2 |  | Living kidney transplantation |
| K780-21 |  | Addition of living donor kidney transplantation (the cost of the donor's nursing care) |
| K780-22 |  | Additional fee for anti-HLA antibody testing (living donor kidney transplantation) |
| **Diseases related to bone density decrease, including osteoporosis** | |  |
| **ICD-10 code** | **ICD-10 name** | **Disease name** |
| A099 | Gastroenteritis and colitis due to unspecified causes | Inflammatory bowel disease |
| B181 | Chronic viral hepatitis B, without delta factor (superinfection) | Decompensated cirrhosis B |
| B182 | Chronic viral hepatitis C | Decompensated cirrhosis C |
| C900 | Multiple myeloma | Multiple myeloma |
| D472 | Monoclonal globulinemia of undetermined significance (MGUS) | Monoclonal gammopathy of undetermined significance |
| E050 | Thyrotoxicosis with diffuse goiter | Basedow's disease |
| E050 | Thyrotoxicosis with diffuse goiter | Recurrence after surgery for Basedow's disease |
| E050 | Thyrotoxicosis with diffuse goiter | Graves' disease |
| E050 | Thyrotoxicosis with diffuse goiter | Euthyroid Graves' disease |
| E050 | Thyrotoxicosis with diffuse goiter | Toxic goiter |
| E050 | Thyrotoxicosis with diffuse goiter | Diffuse toxic goiter |
| E050 | Thyrotoxicosis with diffuse goiter | Basedow's ophthalmopathy |
| E051 | Thyrotoxicosis with toxic solitary thyroid nodules | Toxic mononodular goiter |
| E052 | Thyrotoxicosis with toxic multinodular goiter | Plummer's disease |
| E052 | Thyrotoxicosis with toxic multinodular goiter | Toxic multinodular goiter |
| E053 | Thyrotoxicosis due to ectopic thyroid tissue | Ectopic toxic goiter |
| E054 | Artificial thyrotoxicosis | Artificial thyrotoxicosis |
| E055 | Thyroid storm or acute onset | Thyroid crisis |
| E058 | Other thyrotoxicosis | Pituitary hyperthyroidism |
| E058 | Other thyrotoxicosis | Secondary hyperthyroidism |
| E058 | Other thyrotoxicosis | Pituitary hypersecretion of TSH |
| E059 | Thyrotoxicosis, unspecified | Thyrotoxic exophthalmos |
| E059 | Thyrotoxicosis, unspecified | Transient hyperthyroidism |
| E059 | Thyrotoxicosis, unspecified | Thyrotoxic periodic paralysis |
| E059 | Thyrotoxicosis, unspecified | Primary hyperthyroidism |
| E059 | Thyrotoxicosis, unspecified | Pseudohyperthyroidism |
| E059 | Thyrotoxicosis, unspecified | Thyrotoxic heart failure |
| E059 | Thyrotoxicosis, unspecified | Hyperthyroidism |
| E059 | Thyrotoxicosis, unspecified | Thyrotoxicosis |
| E059 | Thyrotoxicosis, unspecified | Thyrotoxic arthropathy |
| E059 | Thyrotoxicosis, unspecified | Thyrotoxic myasthenia syndrome |
| E059 | Thyrotoxicosis, unspecified | Thyrotoxic cardiomyopathy |
| E059 | Thyrotoxicosis, unspecified | Thyrotoxic quadriplegia |
| E059 | Thyrotoxicosis, unspecified | Thyrotoxic myopathy |
| E059 | Thyrotoxicosis, unspecified | Thyroid ophthalmia |
| E210 | Primary parathyroid <parathyroid> hyperfunction | Parathyroid hyperplasia |
| E210 | Primary parathyroid <parathyroid> hyperfunction | Primary Hyperparathyroidism |
| E211 | Secondary <Secondary> Parathyroid <Parathyroid> Hyperactivity, not otherwise classified | Secondary hyperparathyroidism |
| E213 | Hyperparathyroidism, unspecified | Hyperparathyroidism |
| E213 | Hyperparathyroidism, unspecified | Parathyroid crisis |
| E214 | Other identified parathyroid <parathyroid> disorders | Parathyroid cysts |
| E215 | Parathyroid <Parathyroid> disorder, unspecified | Parathyroid dysfunction |
| E240 | Pituitary-dependent Cushing's disease | ACTH-producing pituitary adenomas |
| E240 | Pituitary-dependent Cushing's disease | Cushing's disease |
| E240 | Pituitary-dependent Cushing's disease | Pituitary-dependent Cushing's disease |
| E240 | Pituitary-dependent Cushing's disease | ACTH-producing tumors |
| E240 | Pituitary-dependent Cushing's disease | Subclinical Cushing's disease |
| E241 | Nelson<Nelson> syndrome | Nelson's syndrome |
| E242 | Drug-induced Cushing syndrome | Drug-induced Cushing's syndrome |
| E243 | Ectopic ACTH (adrenocorticotropic hormone) syndrome | Ectopic ACTH producing tumor |
| E243 | Ectopic ACTH (adrenocorticotropic hormone) syndrome | Ectopic ACTH production syndrome |
| E244 | Alcoholic pseudo-cushing syndrome | Alcoholic pseudo Cushing's syndrome |
| E248 | Other Cushing's syndrome | Adrenocortical nodular hyperplasia |
| E248 | Other Cushing's syndrome | Subclinical cushing syndrome |
| E249 | Cushing's syndrome, unspecified | Cushing's syndrome |
| E274 | Other and unspecified adrenocortical insufficiency (sickness) | Type 4 renal tubular acidosis |
| E283 | Primary ovarian failure (disease) | Luteal dysfunction |
| E283 | Primary ovarian failure (disease) | Early menopause |
| E283 | Primary ovarian failure (disease) | Premature ovarian dysfunction |
| E283 | Primary ovarian failure (disease) | Ovarian deficiency symptom |
| E283 | Primary ovarian failure (disease) | Ovarian hypoplasia |
| E283 | Primary ovarian failure (disease) | Luteal hypofunction syndrome |
| E283 | Primary ovarian failure (disease) | Primary ovarian hypofunction |
| E283 | Primary ovarian failure (disease) | Menopausal ovarian hypofunction |
| E283 | Primary ovarian failure (disease) | Ovarian failure |
| E283 | Primary ovarian failure (disease) | Premature ovarian failure |
| E283 | Primary ovarian failure (disease) | Hypogonadism, female |
| E291 | Testicular (testicular) hypofunction (sickness) | 5-alpha-reductase deficiency |
| E291 | Testicular (testicular) hypofunction (sickness) | Hypogonadism, male |
| E291 | Testicular (testicular) hypofunction (sickness) | Eunuchus |
| E291 | Testicular (testicular) hypofunction (sickness) | Testicular dysfunction |
| E291 | Testicular (testicular) hypofunction (sickness) | Eunuchoid disease |
| E291 | Testicular (testicular) hypofunction (sickness) | Impaired testicular development |
| K529 | Noninfectious gastroenteritis and noninfectious colitis, details unknown | Early-onset inflammatory bowel disease |
| K703 | Alcoholic cirrhosis | Decompensated alcoholic cirrhosis |
| K743 | Primary biliary cirrhosis | Symptomatic primary biliary cirrhosis |
| K746 | Other and unspecified cirrhosis | Decompensated cirrhosis |
| M800 | Postmenopausal osteoporosis with osteoporosis and pathological fractures | Postmenopausal osteoporosis and pathological fracture |
| M8000 | Postmenopausal osteoporosis with osteoporosis and pathologic fractures; multiple sites | Postmenopausal osteoporosis and multiple fractures |
| M8003 | Postmenopausal osteoporosis with osteoporosis and pathologic fractures; forearm | Postmenopausal osteoporosis and forearm fracture |
| M8005 | Postmenopausal osteoporosis with osteoporosis and pathological fractures; pelvis and thigh | Postmenopausal osteoporosis and pathological fracture of pelvis |
| M8005 | Postmenopausal osteoporosis with osteoporosis and pathological fractures; pelvis and thigh | Postmenopausal osteoporosis and pathologic femoral fracture |
| M8006 |  | Postmenopausal osteoporosis and pathological leg fracture |
| M8008 | Postmenopausal osteoporosis (osteoporosis), with pathological fractures; others | Postmenopausal osteoporosis and vertebral fracture |
| M801 | Osteoporosis after oophorectomy (surgery), with pathological fractures | Osteoporosis and pathological fracture after oophorectomy |
| M802 | Disuse osteoporosis <osteoporosis> with pathological fracture | Disuse osteoporosis/pathological fracture |
| M8025 |  | Disuse osteoporosis and pathological fracture of thigh |
| M8028 | Disuse osteoporosis <osteoporosis> with pathological fracture | Disused osteoporosis and vertebral fracture |
| M803 | Postoperative malabsorption osteoporosis with osteoporosis and pathological fracture | Postoperative malabsorption osteoporosis and pathological fracture |
| M804 | Drug-induced osteoporosis (osteoporosis), with pathological fractures | Steroidal osteoporosis, pathological fracture |
| M804 | Drug-induced osteoporosis (osteoporosis), with pathological fractures | Drug-induced osteoporosis with pathological fracture |
| M8040 |  | Steroidal osteoporosis with multiple fractures |
| M8045 | Drug-induced osteoporosis (osteoporosis), with pathological fractures | Steroidal osteoporosis and pelvic pathologic fracture |
| M8045 | Drug-induced osteoporosis (osteoporosis), with pathological fractures | Steroidal osteoporosis and pathological fracture of thigh |
| M8045 | Drug-induced osteoporosis (osteoporosis), with pathological fractures | Drug-induced osteoporosis, femoral pathological fracture |
| M8046 | Drug-induced osteoporosis (osteoporosis), with pathological fractures | Steroidal osteoporosis and pathological leg fracture |
| M8048 | Drug-induced osteoporosis (osteoporosis), with pathological fractures; others | Steroidal vertebral compression fracture |
| M8048 | Drug-induced osteoporosis (osteoporosis), with pathological fractures; others | Steroidal osteoporosis and vertebral fracture |
| M805 | Idiopathic osteoporosis with osteoporosis and pathological fracture | Juvenile osteoporosis with pathological fracture |
| M805 | Idiopathic osteoporosis with osteoporosis and pathological fracture | Idiopathic osteoporosis with pathological fracture |
| M8055 |  | Juvenile osteoporosis and pathological fracture of thigh |
| M8058 | Idiopathic osteoporosis with osteoporosis and pathological fracture | Idiopathic osteoporosis with spontaneous fracture |
| M808 | Other osteoporosis with osteoporosis and pathological fractures | Presence of senile osteoporosis and pathological fracture |
| M8080 | Other osteoporosis with osteoporosis and pathologic fractures; multiple sites | Presence of senile osteoporosis and multiple fractures |
| M8083 | Other osteoporosis with osteoporosis and pathologic fractures; forearm | Presence of osteoporosis senile and forearm fracture |
| M8085 | Other osteoporosis with osteoporosis and pathological fractures; pelvis and thigh | Presence of senile osteoporosis and pathological fracture of pelvis |
| M8085 | Other osteoporosis with osteoporosis and pathological fractures; pelvis and thigh | Presence of senile osteoporosis and pathological fracture of thigh |
| M8086 |  | Presence of senile osteoporosis and pathological leg fracture |
| M8088 | Other osteoporosis with osteoporosis and pathological fractures; others | Presence of senile osteoporosis and vertebral fracture |
| M809 | Osteoporosis with unspecified osteoporosis and pathological fractures | Osteoporosis and pathological fracture |
| M809 | Osteoporosis with unspecified osteoporosis and pathological fractures | Secondary osteoporosis/pathological fracture |
| M8090 | Osteoporosis with unspecified osteoporosis and pathologic fractures; multiple sites | Osteoporosis and multiple fractures |
| M8092 |  | Osteoporosis and brachial fracture |
| M8093 | Osteoporosis with unspecified osteoporosis and pathologic fractures; forearm | Osteoporosis and forearm fracture |
| M8095 | Osteoporosis (osteoporosis), with pathological fractures; pelvis and thigh, unspecified | Osteoporosis and pelvic pathologic fracture |
| M8095 | Osteoporosis (osteoporosis), with pathological fractures; pelvis and thigh, unspecified | Osteoporosis and pathological fracture of thigh |
| M8096 | Osteoporosis with unspecified osteoporosis and pathological fractures | Osteoporosis and pathological fracture of lower leg |
| M8098 | Osteoporosis (osteoporosis), with pathological fractures; others, unspecified | Cervical vertebral osteoporosis and pathological fracture |
| M8098 | Osteoporosis (osteoporosis), with pathological fractures; others, unspecified | Osteoporosis and vertebral fracture |
| M8098 | Osteoporosis (osteoporosis), with pathological fractures; others, unspecified | Vertebral osteoporosis and pathologic fracture |
| M8098 | Osteoporosis (osteoporosis), with pathological fractures; others, unspecified | Osteoporosis and rib fracture |
| M8098 | Osteoporosis (osteoporosis), with pathological fractures; others, unspecified | Secondary osteoporosis and vertebral fracture |
| M8099 | Osteoporosis with unspecified osteoporosis, pathologic fracture; site unknown | Treatment of patients with osteoporosis at high risk for fracture |
| M8099 | Osteoporosis with unspecified osteoporosis, pathologic fracture; site unknown | Severe osteoporosis |
| M8109 | Postmenopausal osteoporosis <osteoporosis>; unknown site | Postmenopausal osteoporosis |
| M8119 | Osteoporosis after ovariectomy (surgery); unknown site | Osteoporosis after oophorectomy |
| M8129 | Disuse osteoporosis <osteoporosis>; unknown site | Disuse osteoporosis |
| M8139 | Postoperative malabsorption osteoporosis <osteoporosis>; unknown site | Postoperative malabsorption osteoporosis |
| M8149 | Drug-induced osteoporosis <osteoporosis>; site unknown | Steroid osteoporosis |
| M8149 | Drug-induced osteoporosis <osteoporosis>; site unknown | Drug-induced osteoporosis |
| M815 | Idiopathic osteoporosis | Idiopathic juvenile osteoporosis |
| M8159 | Idiopathic osteoporosis <osteoporosis>; site unknown | Juvenile osteoporosis |
| M8159 | Idiopathic osteoporosis <osteoporosis>; site unknown | Idiopathic osteoporosis |
| M8168 | Localized osteoporosis <osteoporosis>; others | Vertebral osteoporosis |
| M8168 | Localized osteoporosis <osteoporosis>; others | Cervical vertebral osteoporosis |
| M8189 | Other osteoporosis <osteoporosis>; unknown site | Senile osteoporosis |
| M8199 | Osteoporosis (osteoporosis), unspecified; site unknown | Osteoporosis |
| M8199 | Osteoporosis (osteoporosis), unspecified; site unknown | Secondary osteoporosis |
| N258 | Other disorders resulting from renal tubular dysfunction | Renal acidosis tubular |
| N258 | Other disorders resulting from renal tubular dysfunction | Primary proximal renal tubular acidosis |
| N258 | Other disorders resulting from renal tubular dysfunction | Distal renal tubular acidosis |
| N258 | Other disorders resulting from renal tubular dysfunction | Proximal renal tubular acidosis |
| N258 | Other disorders resulting from renal tubular dysfunction | Primary renal tubular acidosis |
| N258 | Other disorders resulting from renal tubular dysfunction | Secondary proximal renal tubular acidosis |
| N258 | Other disorders resulting from renal tubular dysfunction | Type 1 renal tubular acidosis |
| N258 | Other disorders resulting from renal tubular dysfunction | Type 2 renal tubular acidosis |
| Q822 | Mastocytosis | Mastocytosis |
| **Cancer** |  |  |
| **ICD-10 code** | **ICD-10 name** |  |
| C00-C14 | Malignant neoplasm of lip, oral cavity, and pharynx |  |
| C15-C26 | Malignant neoplasm of digestive tract <tumor> |  |
| C30-C39 | Malignant neoplasm of respiratory and thoracic organs <tumor> |  |
| C40-C41 | Malignant neoplasm of bone and joint cartilage <tumor> |  |
| C43-C44 | Melanoma of the skin and other malignant neoplasms of the skin |  |
| C45-C49 | Malignant neoplasm of mesothelium and soft tissue |  |
| C50 | Malignant neoplasm of breast |  |
| C51-C58 | Malignant neoplasm of female genital tract <tumor> |  |
| C60-C63 | Malignant neoplasm of male genital tract <tumor> |  |
| C64-C68 | Malignant neoplasm of renal urinary tract <tumor> |  |
| C69-C72 | Malignant neoplasm of eye, brain and other central nervous system sites <tumor> |  |
| C73-C75 | Malignant neoplasm of thyroid and other endocrine glands |  |
| C76-C80 | Malignant neoplasm of unknown site, secondary site, or unspecified site |  |
| C81-C96 | Malignant neoplasms of lymphoid, hematopoietic, and associated tissues <tumor>, described or presumed as primary |  |
| C97 | Independent (primary) multisite malignant neoplasm <tumor> |  |
| D00-D09 | Intraepithelial neoplasm <tumor> |  |
| **Parathyroidectomy** | |  |
| **Receipt code** | **Receipt name** |  |
| 150386310 | Endoscopic parathyroid (parathyroid) adenoma hyperplasia surgery |  |
| 150119910 | Parathyroid (Parathyroid) Malignant Tumor Surgery (Extensive) |  |
| 150119810 | Parathyroid (parathyroid) adenoma hyperplasia surgery (total parathyroidectomy, partial muscle transplantation) |  |
| 150119710 | Parathyroid (parathyroid) adenoma hyperplasia surgery (parathyroidectomy) |  |
| 140048150 | Local injection of ethanol (for parathyroid glands) |  |

CAPD, continuous ambulatory peritoneal dialysis; HLA, human leukocyte antigen; TSH, thyroid stimulating hormone

**Online Resource 4.** International Classification of Diseases 10^th^ Revision (ICD-10) codes relevant to the present study (including fracture, mortality, and cardiovascular disease)

| **ICD-10 code** | **ICD-10 name** | **Disease name** |
| --- | --- | --- |
| **Fracture** |  |  |
| S7200 | Femoral neck fracture; closed | Posterior hip dislocation fracture |
| S7200 | Femoral neck fracture; closed | Hip fracture |
| S7200 | Femoral neck fracture; closed | Femoral neck fracture |
| S7200 | Femoral neck fracture; closed | Central dislocation fracture of hip |
| S7200 | Femoral neck fracture; closed | Dislocated hip fracture |
| S7200 | Femoral neck fracture; closed | Trans femoral neck fracture |
| S7200 | Femoral neck fracture; closed | Lateral penetrating femoral neck fracture |
| S7200 | Femoral neck fracture; closed | Lateral femoral neck fracture |
| S7200 | Femoral neck fracture; closed | Basicervical fracture |
| S7200 | Femoral neck fracture; closed | Medial femoral neck fracture |
| S7200 | Femoral neck fracture; closed | Subcapital femoral fracture |
| S7200 | Femoral neck fracture; closed | Femoral head medial fracture |
| S7200 | Femoral neck fracture; closed | Femoral lateral neck fracture |
| S7200 | Femoral neck fracture; closed | Proximal femoral epiphyseal injury |
| S7201 | Femoral neck fracture; open | Femoral neck medial open fracture |
| S7201 | Femoral neck fracture; open | Posterior open dislocation fracture of the hip |
| S7201 | Femoral neck fracture; open | Open fracture of neck of femur |
| S7201 | Femoral neck fracture; open | Open dislocation fracture of hip joint |
| S7201 | Femoral neck fracture; open | Femoral neck transverse open fracture |
| S7201 | Femoral neck fracture; open | Open dislocation fracture of hip |
| S7201 | Femoral neck fracture; open | Open lateral femoral neck fracture |
| S7201 | Femoral neck fracture; open | Open hip fracture |
| S7201 | Femoral neck fracture; open | Open femoral neck fracture |
| S7210 | Transtrochanteric fracture; closed | Trochanteric fracture |
| S7210 | Transtrochanteric fracture; closed | Greater trochanter fracture |
| S7210 | Transtrochanteric fracture; closed | Great trochanteric detachment fracture |
| S7210 | Transtrochanteric fracture; closed | Intertrochanteric fracture |
| S7210 | Transtrochanteric fracture; closed | Pertrochanteric facture |
| S7211 | Transtrochanteric fracture; open | Open greater trochanter fracture |
| S7211 | Transtrochanteric fracture; open | Open femoral trochanteric fracture |
| S7220 | Subtrochanteric fracture; closed | Subtrochanteric fracture |
| S7221 | Subtrochanteric fracture; open | Subtrochanteric open fracture |
| S7230 | Femoral shaft fracture; closed | Femoral shaft fractures |
| S7230 | Femoral shaft fracture; closed | Femoral shaft fracture |
| S7231 | Femoral shaft fracture; open | Open femoral shaft fracture |
| S7231 | Femoral shaft fracture; open | Open fracture of the femur shaft |
| S7240 | Distal femoral fracture; closed | Femoral condyle fracture |
| S7240 | Distal femoral fracture; closed | Distal femoral fracture |
| S7240 | Distal femoral fracture; closed | Supracondylar fracture of the femur |
| S7240 | Distal femoral fracture; closed | External femoral condyle fracture |
| S7240 | Distal femoral fracture; closed | Fracture of the internal condyle of the femur |
| S7240 | Distal femoral fracture; closed | Distal femoral epiphyseal injury |
| S7241 | Distal femoral fracture; open | Distal femoral open fracture |
| S7241 | Distal femoral fracture; open | Open fracture of the internal condyle of the femur |
| S7241 | Distal femoral fracture; open | Supracondylar fracture of femur |
| S7241 | Distal femoral fracture; open | Open fracture of femoral condyle |
| S7241 | Distal femoral fracture; open | Open fracture of external condyle of femur |
| S7270 | Multiple fractures of the femur; closed | Multiple fractures of the femur |
| S7271 | Multiple fractures of the femur; open | Multiple open fracture of femur |
| S7290 | Femoral fracture, unknown site; closed | Femur fracture |
| S7290 | Femoral fracture, unknown site; closed | Femoral insufficiency fracture |
| S7290 | Femoral fracture, unknown site; closed | Crushed femur fracture |
| S7291 | Femoral fracture, unknown site; open | Open fracture of femur |
| S7291 | Femoral fracture, unknown site; open | Femoral crushed open fracture |
| M4849 | Stress fracture of vertebra; unknown site | Vertebral stress fracture |
| M8433 | Stress fractures, n.e.c.; forearm | Ulnar stress fracture |
| M8434 | Stress fractures, not elsewhere classified; hands | First metacarpal stress fracture |
| M8434 | Stress fractures, not elsewhere classified; hands | Stress fracture of metacarpal bone |
| M8435 | Stress fractures, n.e.c.; pelvis and thighs | Ischial stress fracture |
| M8435 | Stress fractures, n.e.c.; pelvis and thighs | Femoral neck stress fracture |
| M8435 | Stress fractures, n.e.c.; pelvis and thighs | Femoral shaft stress fracture |
| M8435 | Stress fractures, n.e.c.; pelvis and thighs | Femoral stress fractures |
| M8435 | Stress fractures, n.e.c.; pelvis and thighs | Pubic stress fracture |
| M8436 | Stress fractures, not elsewhere classified; lower legs | Tibial stress fractures |
| M8436 | Stress fractures, not elsewhere classified; lower legs | Fibular stress fracture |
| M8437 | Stress fractures, not elsewhere classified; ankle and foot | March fracture |
| M8437 | Stress fractures, not elsewhere classified; ankle and foot | Scaphoid stress fracture |
| M8437 | Stress fractures, not elsewhere classified; ankle and foot | Calcaneal stress fracture |
| M8437 | Stress fractures, not elsewhere classified; ankle and foot | Metatarsal Stress Fracture |
| M8438 | Stress fractures, n.e.c. | Rib stress fracture |
| M8439 | Stress fractures, n.e.c.; unknown site | Fatigue periosteal disorder |
| M8439 | Stress fractures, n.e.c.; unknown site | Stress fracture |
| S2200 | Thoracic vertebral fractures; closed | Thoracic vertebral fracture |
| S2200 | Thoracic vertebral fractures; closed | Compression fracture of thoracic vertebra |
| S2200 | Thoracic vertebral fractures; closed | Thoracic dislocation fracture |
| S2200 | Thoracic vertebral fractures; closed | Transverse process fracture of chest |
| S2200 | Thoracic vertebral fractures; closed | Thoracic vertebral fracture |
| S2200 | Thoracic vertebral fractures; closed | Thoracic spinous process fracture |
| S2200 | Thoracic vertebral fractures; closed | Ruptured thoracic vertebra fracture |
| S2200 | Thoracic vertebral fractures; closed | Transverse process fracture of thoracic vertebra |
| S2200 | Thoracic vertebral fractures; closed | Thoracic arch fracture |
| S2200 | Thoracic vertebral fractures; closed | Thoracic vertebral fracture |
| S2200 | Thoracic vertebral fractures; closed | Subclinical thoracic fracture |
| S2201 | Thoracic vertebral fractures; open | Open thoracic dislocation fracture |
| S2201 | Thoracic vertebral fractures; open | Open fracture of thoracic vertebra |
| S2201 | Thoracic vertebral fractures; open | Open thoracic vertebral transverse process fracture |
| S2201 | Thoracic vertebral fractures; open | Open thoracic arch fracture |
| S2201 | Thoracic vertebral fractures; open | Open thoracic spinous process fracture |
| S2201 | Thoracic vertebral fractures; open | Open fracture of thoracic vertebral body |
| S2210 | Multiple fractures of thoracic vertebra; closed | Multiple thoracic fractures |
| S2210 | Multiple fractures of thoracic vertebra; closed | Multiple thoracic fractures |
| S2210 | Multiple fractures of thoracic vertebra; closed | Multiple compression fracture of thoracic vertebra |
| S2211 | Multiple fractures of thoracic vertebra; open | Multiple open thoracic vertebral fractures |
| S2220 | Sternal fracture; closed | Sternal fracture |
| S2220 | Sternal fracture; closed | Fracture of pedicle of sternum |
| S2220 | Sternal fracture; closed | Sternal fissure fracture |
| S2220 | Sternal fracture; closed | Sternal insufficiency fracture |
| S2221 | Breakage of sternum; open | Open sternal fracture |
| S2230 | Rib fracture; closed | Rib fracture |
| S2230 | Rib fracture; closed | Rib insufficiency fracture |
| S2230 | Rib fracture; closed | Costochondral fracture |
| S2230 | Rib fracture; closed | Inapparent rib fracture |
| S2231 | Rib fracture; open | Open fracture of rib |
| S2240 | Multiple rib fractures; closed | Rib fissure fracture |
| S2240 | Multiple rib fractures; closed | Multiple rib fractures |
| S2241 | Multiple rib fractures; open | Multiple open fracture of rib |
| S320 | Lumbar vertebral fracture | Lumbar spine injury |
| S3200 | Lumbar vertebral fractures; occlusive | Lumbar compression fracture |
| S3200 | Lumbar vertebral fractures; occlusive | Lumbar dislocation fracture |
| S3200 | Lumbar vertebral fractures; occlusive | Lumbar vertebral fracture |
| S3200 | Lumbar vertebral fractures; occlusive | Lumbar transverse process fracture |
| S3200 | Lumbar vertebral fractures; occlusive | Lumbar spinous process fracture |
| S3200 | Lumbar vertebral fractures; occlusive | Lumbar insufficiency fracture |
| S3200 | Lumbar vertebral fractures; occlusive | Lumbar rupture fracture |
| S3200 | Lumbar vertebral fractures; occlusive | Lumbar vertebral arch fracture |
| S3200 | Lumbar vertebral fractures; occlusive | Lumbar vertebral fracture |
| S3200 | Lumbar vertebral fractures; occlusive | Subclinical lumbar fracture |
| S3201 | Lumbar vertebral fractures; open | Open fracture of lumbar spine |
| S3201 | Lumbar vertebral fractures; open | Open lumbar transverse process fracture |
| S3201 | Lumbar vertebral fractures; open | Open lumbar vertebral fracture |
| S3210 | Sacral fracture; closed | Crack fracture of sacrum |
| S3210 | Sacral fracture; closed | Fracture of sacrum |
| S3211 | Sacral fracture; open | Open fracture of sacrum |
| S3220 | Coccygeal fracture; closed | Coccygeal fracture |
| S3221 | Fracture of coccyx; open | Open fracture of coccyx |
| S3230 | Iliac fracture; closed | Iliac fracture |
| S3230 | Iliac fracture; closed | Iliac abrasion fracture |
| S3230 | Iliac fracture; closed | Anterior inferior iliac spine detachment fracture |
| S3230 | Iliac fracture; closed | Anterior superior iliac spine detachment fracture |
| S3231 | Iliac fracture; open | Open fracture of ilium |
| S3240 | Acetabular fracture; occlusive | Acetabulum fracture |
| S3241 | Acetabular fracture; open | Open fracture of acetabulum |
| S3250 | Pubic fracture; closed | Pubic fracture |
| S3251 | Pubic fracture; open | Open pubic fracture |
| S3270 | Multiple lumbar and pelvic fractures; closed | Double vertical pelvic fracture |
| S3270 | Multiple lumbar and pelvic fractures; closed | Malgaigne fracture |
| S3270 | Multiple lumbar and pelvic fractures; closed | Multiple pelvic fractures |
| S3270 | Multiple lumbar and pelvic fractures; closed | Multiple lumbar pelvic fractures |
| S3270 | Multiple lumbar and pelvic fractures; closed | Multiple lumbar compression fractures |
| S3270 | Multiple lumbar and pelvic fractures; closed | Multiple lumbar vertebral fractures |
| S3271 | Multiple lumbar and pelvic fractures; open | Multiple vertical pelvic open fractures |
| S3280 | Other and unspecified fractures of lumbar spine and pelvis; closed | Pelvic fracture |
| S3280 | Other and unspecified fractures of lumbar spine and pelvis; closed | Sciatic tubercle avulsion fracture |
| S3280 | Other and unspecified fractures of lumbar spine and pelvis; closed | Ischial fracture |
| S3280 | Other and unspecified fractures of lumbar spine and pelvis; closed | Lumbosacral fracture |
| S3280 | Other and unspecified fractures of lumbar spine and pelvis; closed | Ring fracture |
| S3281 | Fractures of lumbar spine and pelvis other and unspecified sites; open | Open fracture of pelvis |
| S3281 | Fractures of lumbar spine and pelvis other and unspecified sites; open | Open lumbosacral fracture |
| S3281 | Fractures of lumbar spine and pelvis other and unspecified sites; open | Open fracture of ischium |
| S3281 | Fractures of lumbar spine and pelvis other and unspecified sites; open | Open fracture of pelvic ring |
| S4200 | Clavicular fracture; closed | Fracture of clavicle |
| S4200 | Clavicular fracture; closed | Distal clavicle fracture |
| S4200 | Clavicular fracture; closed | Clavicular fracture |
| S4200 | Clavicular fracture; closed | Fracture of acromion |
| S4200 | Clavicular fracture; closed | Clavicular metaphysis fracture |
| S4200 | Clavicular fracture; closed | Clavicular fracture |
| S4201 | Fracture of clavicle; open | Open fracture of clavicle |
| S4201 | Fracture of clavicle; open | Open fracture of acromion |
| S4201 | Fracture of clavicle; open | Open fracture of clavicle shaft |
| S4201 | Fracture of clavicle; open | Open fracture of distal clavicle |
| S4210 | Fracture of scapula; closed | Shoulder dislocation fracture |
| S4210 | Fracture of scapula; closed | Scapuloacromial fracture |
| S4210 | Fracture of scapula; closed | Fracture of scapula |
| S4210 | Fracture of scapula; closed | Scapular coracoid fracture |
| S4210 | Fracture of scapula; closed | Scapular glenoid fossa fracture |
| S4210 | Fracture of scapula; closed | Fracture of scapular neck |
| S4210 | Fracture of scapula; closed | Fracture of body of scapula |
| S4211 | Fracture of scapula; open | Open dislocation fracture of shoulder joint |
| S4211 | Fracture of scapula; open | Open scapular coracoid fracture |
| S4211 | Fracture of scapula; open | Open fracture of scapula |
| S4211 | Fracture of scapula; open | Open fracture of scapular glenoid fossa |
| S4211 | Fracture of scapula; open | Open scapula-acromion fracture |
| S4220 | Proximal humerus fracture; closed | Humeral surgical neck fracture |
| S4220 | Proximal humerus fracture; closed | Humeral head fracture |
| S4220 | Proximal humerus fracture; closed | Fracture of neck of humerus |
| S4220 | Proximal humerus fracture; closed | Humeral anatomic neck fracture |
| S4220 | Proximal humerus fracture; closed | Proximal humerus fracture |
| S4220 | Proximal humerus fracture; closed | Fracture of the proximal humerus |
| S4220 | Proximal humerus fracture; closed | Fracture of cervical dislocation of humerus |
| S4220 | Proximal humerus fracture; closed | Greater tubercle fracture of humerus |
| S4220 | Proximal humerus fracture; closed | Avulsion fracture of major tubercle of humerus |
| S4220 | Proximal humerus fracture; closed | Injury to proximal epiphysis of humerus |
| S4220 | Proximal humerus fracture; closed | Humeral nodular fracture |
| S4221 | Fracture of the proximal humerus; open | Open fracture of neck of humerus |
| S4221 | Fracture of the proximal humerus; open | Open fracture of the proximal humerus |
| S4221 | Fracture of the proximal humerus; open | Open fracture of humeral head |
| S4221 | Fracture of the proximal humerus; open | Open fracture of proximal humerus |
| S4221 | Fracture of the proximal humerus; open | Cervical opening fracture of humerus |
| S4230 | Fracture of metaphysis of humerus; closed | Humerus fracture |
| S4230 | Fracture of metaphysis of humerus; closed | Shaft fracture of humerus |
| S4230 | Fracture of metaphysis of humerus; closed | Humeral dislocation fracture |
| S4230 | Fracture of metaphysis of humerus; closed | Fracture of humerus |
| S4230 | Fracture of metaphysis of humerus; closed | Spiral fracture of humerus |
| S4231 | Fracture of humerus shaft; open | Open fracture of humerus |
| S4231 | Fracture of humerus shaft; open | Open fracture of humerus |
| S4231 | Fracture of humerus shaft; open | Open dislocation fracture of humerus |
| S4231 | Fracture of humerus shaft; open | Open fracture of humerus shaft |
| S4240 | Fracture of the distal humerus; closed | Fracture of lateral condyle of humerus |
| S4240 | Fracture of the distal humerus; closed | Fracture of epicondyle of humerus |
| S4240 | Fracture of the distal humerus; closed | Fracture of intrahumeral condyle |
| S4240 | Fracture of the distal humerus; closed | Supracondylar fracture of the humerus |
| S4240 | Fracture of the distal humerus; closed | Fracture of condyle of humerus |
| S4240 | Fracture of the distal humerus; closed | Fracture of lateral epicondyle of humerus |
| S4240 | Fracture of the distal humerus; closed | Fractures of the distal humerus |
| S4240 | Fracture of the distal humerus; closed | Distal humerus fracture |
| S4240 | Fracture of the distal humerus; closed | Intercondylar fracture of humerus |
| S4240 | Fracture of the distal humerus; closed | Fracture of condyle of humerus |
| S4240 | Fracture of the distal humerus; closed | Capitellum fracture |
| S4240 | Fracture of the distal humerus; closed | Injury to the distal epiphysis of the humerus |
| S4240 | Fracture of the distal humerus; closed | Transcondylar fracture |
| S4240 | Fracture of the distal humerus; closed | Humeral trochlear fracture |
| S4241 | Fracture of distal humerus; open | Fracture of the epicondyle of the humerus |
| S4241 | Fracture of distal humerus; open | Open fracture of the distal humerus |
| S4241 | Fracture of distal humerus; open | Fracture of open condyle of humerus |
| S4241 | Fracture of distal humerus; open | Fracture of the intrahumeral condyle |
| S4241 | Fracture of distal humerus; open | Supracondylar fracture |
| S4241 | Fracture of distal humerus; open | Small head opening fracture of humerus |
| S4241 | Fracture of distal humerus; open | Open fracture of lateral condyle of humerus |
| S4241 | Fracture of distal humerus; open | Open fracture of distal humerus |
| S4241 | Fracture of distal humerus; open | Fracture of the lateral epicondyle of the humerus |
| S4241 | Fracture of distal humerus; open | Open fracture of condyle of humerus |
| S4241 | Fracture of distal humerus; open | Open humeral condyle fracture |
| S4290 | Fractures of the scapula <upper extremity> band, unknown site; occlusive | Shoulder fracture |
| S4291 | Fractures of the scapula <upper extremity> band, unknown site; open | Open fracture of shoulder |
| S5200 | Ulnar proximal end fracture; closed | Elbow fracture |
| S5200 | Ulnar proximal end fracture; closed | Dislocation fracture of elbow |
| S5200 | Ulnar proximal end fracture; closed | Olecranon fracture |
| S5200 | Ulnar proximal end fracture; closed | Ulnar elbow fracture |
| S5200 | Ulnar proximal end fracture; closed | Proximal ulna fracture |
| S5200 | Ulnar proximal end fracture; closed | Ulnar uncinate process fracture |
| S5200 | Ulnar proximal end fracture; closed | Monteggia fracture |
| S5200 | Ulnar proximal end fracture; closed | Intraelbow fracture |
| S5200 | Ulnar proximal end fracture; closed | Olecephalic fracture |
| S5200 | Ulnar proximal end fracture; closed | Ulnar proximal epiphyseal injury |
| S5201 | Ulnar proximal end fracture; open | Open fracture of proximal ulna |
| S5201 | Ulnar proximal end fracture; open | Ulnar olecranon fracture |
| S5201 | Ulnar proximal end fracture; open | Open dislocation fracture of elbow |
| S5201 | Ulnar proximal end fracture; open | Open fracture of uncinate process of ulna |
| S5201 | Ulnar proximal end fracture; open | Open elbow fracture |
| S5210 | Fractures of the proximal radius; occlusive | Proximal radius fracture |
| S5210 | Fractures of the proximal radius; occlusive | Fracture of radial neck |
| S5210 | Fractures of the proximal radius; occlusive | Fractured radial head |
| S5210 | Fractures of the proximal radius; occlusive | Radial head fracture |
| S5211 | Fractures of the proximal radius; open | Open fracture of radial head |
| S5211 | Fractures of the proximal radius; open | Open fracture of radial neck |
| S5211 | Fractures of the proximal radius; open | Open fracture of proximal radius |
| S5220 | Ulnar metaphyseal fracture; closed | Fracture of ulna |
| S5220 | Ulnar metaphyseal fracture; closed | Ulnar metaphysis fracture |
| S5220 | Ulnar metaphyseal fracture; closed | Ulnar crack fracture |
| S5221 | Ulnar metaphyseal fracture; open | Open fracture of shaft of ulna |
| S5221 | Ulnar metaphyseal fracture; open | Open fracture of ulna |
| S5230 | Fractures of the metaphysis of the radius; closed | Radial metaphysis fracture |
| S5230 | Fractures of the metaphysis of the radius; closed | Fracture of radius |
| S5231 | Fractures of the radial metaphysis; open | Open fracture of radial shaft |
| S5240 | Fractures of both shafts of the ulna and radius; closed | Fracture of radial ulna |
| S5240 | Fractures of both shafts of the ulna and radius; closed | Fracture of the metaphysis of the radial ulna |
| S5241 | Fractures of both shafts of the ulna and radius; open | Open fracture of radial ulna |
| S5241 | Fractures of both shafts of the ulna and radius; open | Open fracture of radial ulna shaft |
| S5250 | Fracture of distal radius; occlusive | Smith fracture |
| S5250 | Fracture of distal radius; occlusive | Galeazzi fracture |
| S5250 | Fracture of distal radius; occlusive | Burton fracture |
| S5250 | Fracture of distal radius; occlusive | Cores fracture |
| S5250 | Fracture of distal radius; occlusive | Fracture of the distal radius |
| S5250 | Fracture of distal radius; occlusive | Fractured radial styloid process |
| S5250 | Fracture of distal radius; occlusive | Distal radial epiphyseal injury |
| S5250 | Fracture of distal radius; occlusive | Distal radius fracture |
| S5250 | Fracture of distal radius; occlusive | Fracture of the distal radius |
| S5251 | Fracture of distal radius; open | Open fracture of the distal radius |
| S5251 | Fracture of distal radius; open | Open fracture of radial styloid process |
| S5251 | Fracture of distal radius; open | Open fracture of the distal radius |
| S5260 | Fractures of the distal ends of the ulna and radius; closed | Fracture of the distal radial ulna |
| S5260 | Fractures of the distal ends of the ulna and radius; closed | Fracture of the distal end of the radial ulna |
| S5261 | Fractures of the distal ends of the ulna and radius; open | Open fracture of the distal radial ulna |
| S5261 | Fractures of the distal ends of the ulna and radius; open | Open distal end fracture of radial ulna |
| S5270 | Multiple fractures of forearm; closed | Multiple fractures of forearm |
| S5271 | Multiple fractures of forearm; open | Multiple open fracture of forearm |
| S5280 | Fractures in other parts of the forearm; closed | Fracture of radius |
| S5280 | Fractures in other parts of the forearm; closed | Distal ulnar fracture |
| S5280 | Fractures in other parts of the forearm; closed | Fractured ulnar styloid process |
| S5280 | Fractures in other parts of the forearm; closed | Ulnar head fracture |
| S5281 | Fractures in other parts of the forearm; open | Open ulnar head fracture |
| S5281 | Fractures in other parts of the forearm; open | Open fracture of radius |
| S5281 | Fractures in other parts of the forearm; open | Open fracture of distal ulna |
| S5281 | Fractures in other parts of the forearm; open | Open fracture of the styloid process of ulna |
| S5290 | Fracture of forearm, unknown site; occlusive | Fracture of forearm |
| S5290 | Fracture of forearm, unknown site; occlusive | Junior wooden fracture of forearm |
| S5291 | Fracture of forearm, unknown site; open | Open forearm fracture |
| S8200 | Patella fracture; closed | Patella fracture |
| S8200 | Patella fracture; closed | Knee fracture |
| S8200 | Patella fracture; closed | Knee dislocation fracture |
| S8200 | Patella fracture; closed | Intraknee fracture |
| S8200 | Patella fracture; closed | Patellar insufficiency fracture |
| S8200 | Patella fracture; closed | Crushed patella fracture |
| S8200 | Patella fracture; closed | Subclinical fracture of patella |
| S8201 | Patella fracture; open | Open dislocation fracture of knee joint |
| S8201 | Patella fracture; open | Open patellar fracture |
| S8201 | Patella fracture; open | Open knee fracture |
| S8201 | Patella fracture; open | Open patella fracture |
| S8210 | Proximal tibial end fracture; closed | Tibial plateau fracture |
| S8210 | Proximal tibial end fracture; closed | Tibial eminence fracture |
| S8210 | Proximal tibial end fracture; closed | Tibial condyle fracture |
| S8210 | Proximal tibial end fracture; closed | Tibial condyle detachment fracture |
| S8210 | Proximal tibial end fracture; closed | Tibial condylar fracture |
| S8210 | Proximal tibial end fracture; closed | Avulsion fracture of tibial lateral condyle |
| S8210 | Proximal tibial end fracture; closed | Proximal tibial end fracture |
| S8210 | Proximal tibial end fracture; closed | Proximal tibial fracture |
| S8210 | Proximal tibial end fracture; closed | Tibial tubercle avulsion fracture |
| S8210 | Proximal tibial end fracture; closed | Intra-tibial knee fracture |
| S8210 | Proximal tibial end fracture; closed | Tibial tuberosity fracture |
| S8210 | Proximal tibial end fracture; closed | Posterior cruciate adhesive detachment fracture |
| S8210 | Proximal tibial end fracture; closed | Anterior cruciate adhesive detachment fracture |
| S8210 | Proximal tibial end fracture; closed | Proximal end fracture of tibial fibula |
| S8210 | Proximal tibial end fracture; closed | Proximal tibial fibular fracture |
| S8210 | Proximal tibial end fracture; closed | Tibial proximal epiphysis injury |
| S8210 | Proximal tibial end fracture; closed | Asymptomatic fracture of the proximal tibia |
| S8211 | Tibial proximal end fracture; open | Open fracture of tibial tuberosity |
| S8211 | Tibial proximal end fracture; open | Open tibial condyle fracture |
| S8211 | Tibial proximal end fracture; open | Open fracture of tibial plateau |
| S8211 | Tibial proximal end fracture; open | Open tibial condyle fracture |
| S8211 | Tibial proximal end fracture; open | Open tibial proximal end fracture |
| S8211 | Tibial proximal end fracture; open | Open fracture of the proximal tibia |
| S8211 | Tibial proximal end fracture; open | Open fracture of the tibial eminence |
| S8211 | Tibial proximal end fracture; open | Open fracture of proximal tibial fibula |
| S8211 | Tibial proximal end fracture; open | Open fracture of the proximal end of the tibia fibula |
| S8220 | Tibial shaft fracture; closed | Tibial fracture |
| S8220 | Tibial shaft fracture; closed | Diaphyseal tibial fractures |
| S8220 | Tibial shaft fracture; closed | Tibial fracture |
| S8220 | Tibial shaft fracture; closed | Tibial fibula fracture |
| S8220 | Tibial shaft fracture; closed | Tibial fibular shaft fracture |
| S8220 | Tibial shaft fracture; closed | Tibial fibular shaft fracture |
| S8220 | Tibial shaft fracture; closed | Subclinical fracture of tibia |
| S8221 | Tibial shaft fracture; open | Open fracture of tibial fibula |
| S8221 | Tibial shaft fracture; open | Open fracture of tibial shaft |
| S8221 | Tibial shaft fracture; open | Open tibial fracture |
| S8221 | Tibial shaft fracture; open | Open tibia fracture |
| S8221 | Tibial shaft fracture; open | Open fracture of tibial fibula shaft |
| S8221 | Tibial shaft fracture; open | Open fracture of tibial fibula shaft |
| S8230 | Distal tibial end fracture; closed | Distal tibial end fracture |
| S8230 | Distal tibial end fracture; closed | Tibial distal epiphyseal injury |
| S8230 | Distal tibial end fracture; closed | Tibial skull fracture |
| S8230 | Distal tibial end fracture; closed | Distal tibial fibular fracture |
| S8230 | Distal tibial end fracture; closed | Distal tibial fibular fracture |
| S8231 | Distal tibial fracture; open | Open fracture of the distal tibia |
| S8231 | Distal tibial fracture; open | Open tibial skull fracture |
| S8231 | Distal tibial fracture; open | Open fracture of the distal tibial fibula |
| S8231 | Distal tibial fracture; open | Open fracture of the distal tibial fibula |
| S8240 | Peroneal-only fracture; closed | Fibular crack fracture |
| S8240 | Peroneal-only fracture; closed | Fibular fracture |
| S8240 | Peroneal-only fracture; closed | Peroneal avulsion fracture |
| S8240 | Peroneal-only fracture; closed | Fracture of the diaphysis of the fibula |
| S8240 | Peroneal-only fracture; closed | Fibular head fracture |
| S8240 | Peroneal-only fracture; closed | Fracture of the fibular head |
| S8240 | Peroneal-only fracture; closed | Distal end fracture of fibula |
| S8240 | Peroneal-only fracture; closed | Distal end of fibula avulsion fracture |
| S8240 | Peroneal-only fracture; closed | Proximal end fracture of fibula |
| S8240 | Peroneal-only fracture; closed | Proximal fibular epiphyseal injury |
| S8241 | Peroneal-only fracture; open | Open fracture of fibula |
| S8241 | Peroneal-only fracture; open | Open fracture of fibula |
| S8241 | Peroneal-only fracture; open | Open fracture of shaft of fibula |
| S8241 | Peroneal-only fracture; open | Open fracture of proximal end of fibula |
| S8241 | Peroneal-only fracture; open | Open fracture of head of fibula |
| S8241 | Peroneal-only fracture; open | Open fracture of the distal end of fibula |
| S8250 | Mesenchymal fracture; closed | Internal malleolar fracture of ankle |
| S8250 | Mesenchymal fracture; closed | Posterior malleolar fracture of ankle |
| S8251 | Mesenchymal fracture; open | Open malleolar fracture of ankle |
| S8251 | Mesenchymal fracture; open | Open posterior malleolar fracture of ankle |
| S8260 | External malleolar fracture; closed | Dupuytren fracture |
| S8260 | External malleolar fracture; closed | Lateral malleolar fracture of ankle |
| S8260 | External malleolar fracture; closed | Exfoliation fracture of ankle |
| S8260 | External malleolar fracture; closed | Peroneal distal epiphyseal injury |
| S8261 | External malleolar fracture; open | Open lateral malleolar fracture of ankle |
| S8261 | External malleolar fracture; open | Exfoliation open fracture of ankle joint |
| S8270 | Multiple fractures of the lower leg; closed | Multiple fractures of lower leg |
| S8270 | Multiple fractures of the lower leg; closed | Proximal end of tibia fracture |
| S8271 | Multiple fractures of the lower leg; open | Open fracture of proximal end of tibia |
| S8271 | Multiple fractures of the lower leg; open | Multiple open fracture of lower leg |
| S8280 | Fractures in other parts of the lower leg; occlusive | Ankle fractures |
| S8280 | Fractures in other parts of the lower leg; occlusive | Fracture‐dislocation of ankle |
| S8280 | Fractures in other parts of the lower leg; occlusive | Malleolar fracture |
| S8280 | Fractures in other parts of the lower leg; occlusive | Trimalleolar fracture |
| S8280 | Fractures in other parts of the lower leg; occlusive | Ankle anterior tubercle fracture |
| S8280 | Fractures in other parts of the lower leg; occlusive | Ankle dislocation fracture |
| S8280 | Fractures in other parts of the lower leg; occlusive | Bilateral malleolar fracture |
| S8280 | Fractures in other parts of the lower leg; occlusive | Intra-ankle fracture |
| S8281 | Fractures in other parts of the lower leg; open | Open dislocation fracture of ankle |
| S8281 | Fractures in other parts of the lower leg; open | Open fracture of bilateral malleolus |
| S8281 | Fractures in other parts of the lower leg; open | Ankle dislocation open fracture |
| S8281 | Fractures in other parts of the lower leg; open | Open malleolus fracture |
| S8281 | Fractures in other parts of the lower leg; open | Open fracture of ankle |
| S8281 | Fractures in other parts of the lower leg; open | Open trimalleolar ankle fracture |
| S8281 | Fractures in other parts of the lower leg; open | Open dislocation fracture of ankle |
| T0210 | Those with fractures of the chest <cage> or of the lower back and pelvis | Compression fracture of thoracolumbar spine |
| T0220 | First cervical vertebral fracture; occlusive | Multiple fractures of one arm |
| T0220 | First cervical vertebral fracture; occlusive | Fracture of brachial forearm |
| T0220 | First cervical vertebral fracture; occlusive | Fracture of carpal bone at the radial end |
| T0221 | First cervical vertebral fracture; open | Multiple open fractures of one arm |
| T0221 | First cervical vertebral fracture; open | Open fracture of brachial forearm |
| T0230 | Multiple fractures of one lower limb; occlusive | Multiple fractures of one lower limb |
| T0231 | Multiple fractures of one lower limb; open | Multiple open fractures of one lower limb |
| T0240 | Multiple fractures of both upper extremities; closed | Multiple bilateral upper extremity fractures |
| T0241 | Multiple fractures of both upper extremities; open | Bilateral upper limb multiple open fracture |
| T0250 | Multiple fractures of both lower limbs; closed | Multiple bilateral lower limb fractures |
| T0251 | Multiple fractures of both lower limbs; open | Multiple open fractures of both lower limbs |
| T0260 | Multiple fractures of the upper extremities, with fractures of the lower extremities; closed | Multiple upper limb fractures and lower limb fractures |
| T0280 | Other composite fractures; closed | Composite fracture |
| T0281 | Other composite fractures; open | Open fracture of the combined site |
| T0290 | Multiple fractures, unspecified; closed | Multiple fractures |
| T0290 | Multiple fractures, unspecified; closed | Multiple lower limb fractures |
| T0291 | Multiple fractures, unspecified; open | Multiple complex fractures |
| T0291 | Multiple fractures, unspecified; open | Multiple open fracture |
| T0291 | Multiple fractures, unspecified; open | Multiple open fracture of lower extremity |
| T08-0 | Vertebral fractures, unspecified; closed | Chance fracture |
| T08-0 | Vertebral fractures, unspecified; closed | Seat belt fracture |
| T08-0 | Vertebral fractures, unspecified; closed | Spinal compression fracture |
| T08-0 | Vertebral fractures, unspecified; closed | Vertebral fracture |
| T08-0 | Vertebral fractures, unspecified; closed | Vertebral arch fracture |
| T08-0 | Vertebral fractures, unspecified; closed | Collapsed vertebra |
| T08-0 | Vertebral fractures, unspecified; closed | Vertebral fracture |
| T08-0 | Vertebral fractures, unspecified; closed | Vertebral dislocation fracture |
| T08-0 | Vertebral fractures, unspecified; closed | Posterior element fracture |
| T08-0 | Vertebral fractures, unspecified; closed | Fractured transverse process |
| T08-0 | Vertebral fractures, unspecified; closed | Spinous processes fractures |
| T08-0 | Vertebral fractures, unspecified; closed | Vertebral horn transection |
| T08-1 | Vertebral fracture, unspecified; open | Open fracture of transverse process |
| T10-0 | Upper limb fracture, unknown site; occlusive | Fracture of upper extremity |
| T10-1 | Upper limb fracture, unknown site; open | Open fracture of upper extremity |
| T1420 | Unlocated fracture; closed | Woody fracture |
| T1420 | Unlocated fracture; closed | Transverse fracture |
| T1420 | Unlocated fracture; closed | Longitudinal fracture |
| T1420 | Unlocated fracture; closed | Double fracture |
| T1420 | Unlocated fracture; closed | Ruptured fracture |
| T1420 | Unlocated fracture; closed | Complete fracture |
| T1420 | Unlocated fracture; closed | Joint fracture |
| T1420 | Unlocated fracture; closed | Joint dislocation fracture |
| T1420 | Unlocated fracture; closed | Crack fracture |
| T1420 | Unlocated fracture; closed | Fractures |
| T1420 | Unlocated fracture; closed | Sesamoid fracture |
| T1420 | Unlocated fracture; closed | Linear fracture |
| T1420 | Unlocated fracture; closed | Dislocation fracture |
| T1420 | Unlocated fracture; closed | Simple fracture |
| T1420 | Unlocated fracture; closed | Detachment fracture |
| T1420 | Unlocated fracture; closed | Insufficiency fracture |
| T1420 | Unlocated fracture; closed | Cleft fracture |
| T1420 | Unlocated fracture; closed | Spiral fracture |
| T1420 | Unlocated fracture; closed | Closed fracture |
| T1420 | Unlocated fracture; closed | Flexion fracture |
| T1420 | Unlocated fracture; closed | Compression fracture |
| T1420 | Unlocated fracture; closed | Retracted fracture |
| T1420 | Unlocated fracture; closed | Oblique fracture |
| T1420 | Unlocated fracture; closed | Dislocated fracture |
| T1420 | Unlocated fracture; closed | Crushed fracture |
| T1420 | Unlocated fracture; closed | Dehiscence fracture |
| T1420 | Unlocated fracture; closed | Intra-articular fracture |
| T1420 | Unlocated fracture; closed | Subclinical fracture |
| T1421 | Fractures of unknown location; open | Complicated fracture |
| T1421 | Fractures of unknown location; open | Open fracture |
| T1421 | Fractures of unknown location; open | Open dislocation fracture |
| T1421 | Fractures of unknown location; open | Open fracture |
| T1421 | Fractures of unknown location; open | Open retracted fracture |
| **Mortality** |  |  |
| G938 | Other well-defined injurious visceral sudden death <sudden death> in the brain | State of brain death |
| I461 | Sudden cardiac death <sudden death> | Sudden cardiac death |
| I469 | Cardiac arrest, unspecified | Cardiac arrest |
| I469 | Cardiac arrest, unspecified | Cardiopulmonary arrest |
| I469 | Cardiac arrest, unspecified | Visit cardiopulmonary arrest |
| R960 | Instantaneous death | Instantaneous death |
| R960 | Instantaneous death | Sudden death |
| R98 | Death without witness | Corpse found |
| R99 | Other deaths for which the diagnosis name is unclear and for unknown cause | Death unexplained |
| **Cardiovascular disease** | |  |
| I489 | Unspecified atrial fibrillation and atrial flutter |  |
| I509 | Congestive Heart Failure |  |
| I639 | Cerebral infarction, unspecified |  |
| I209 | Angina Pectoris, NOS |  |
| I210 | Acute transmural myocardial infarction of anterior wall |  |
| I213 | Myocardial Infarction, Acute, Unspecified (initial episode of care) |  |

NOS, not otherwise specified

**Online Resource 5.** Supplementary text

Comorbidities included in the multivariable regression analysis included the following: hypertension, dyslipidemia, hyperuricemia, diabetes, alcohol dependence, rheumatoid arthritis, dementia, sleep disorder, chronic obstructive pulmonary disease, and fracture. Prescribed medications included as covariates included the following: oral glucocorticoids, proton pump inhibitors, hormone replacement therapy drugs, thiazolidinedione antidiabetic drugs, oral beta-blockers, oral loop diuretics, warfarin, sleep aids, anxiolytics, antiepileptic drugs, selective serotonin reuptake inhibitors, calcium-sensing receptor agonists, and phosphate-binding agents.
